# Bacteria associated with cockroaches: health risk or biotechnological opportunity?

**DOI:** 10.1007/s00253-020-10973-6

**Published:** 2020-10-31

**Authors:** Juan Guzman, Andreas Vilcinskas

**Affiliations:** 1 Department of Bioresources, Fraunhofer Institute for Molecular Biology and Applied Ecology, Ohlebergsweg 12, 35392 Giessen, Germany; 2 Institute for Insect Biotechnology, Justus-Liebig-University of Giessen, Heinrich-Buff-Ring 26-32, 35392 Giessen, Germany

**Keywords:** Blattodea, Cockroach, Microbiome, Bacteria, Pathogens, Application, Biotechnology

## Abstract

**Abstract:**

Cockroaches have existed for 300 million years and more than 4600 extant species have been described. Throughout their evolution, cockroaches have been associated with bacteria, and today *Blattabacterium* species flourish within specialized bacteriocytes, recycling nitrogen from host waste products. Cockroaches can disseminate potentially pathogenic bacteria via feces and other deposits, particularly members of the family *Enterobacteriaceae*, but also *Staphylococcus* and *Mycobacterium* species, and thus, they should be cleared from sites where hygiene is essential, such as hospitals and kitchens. On the other hand, cockroaches also carry bacteria that may produce metabolites or proteins with potential industrial applications. For example, an antibiotic-producing *Streptomyces* strain was isolated from the gut of the American cockroach *Periplaneta americana*. Other cockroach-associated bacteria, including but not limited to *Bacillus*, *Enterococcus*, and *Pseudomonas* species, can also produce bioactive metabolites that may be suitable for development as pharmaceuticals or plant protection products. Enzymes that degrade industrially relevant substrates, or that convert biomasses into useful chemical precursors, are also expressed in cockroach-derived bacteria and could be deployed for use in the food/feed, paper, oil, or cosmetics industries. The analysis of cockroach gut microbiomes has revealed a number of lesser-studied bacteria that may form the basis of novel taxonomic groups. Bacteria associated with cockroaches can therefore be dangerous or useful, and this review explores the bacterial clades that may provide opportunities for biotechnological exploitation.

**Key points:**

• *Members of the Enterobacteriaceae are the most frequently cultivated bacteria from cockroaches.*

*• Cultivation-independent studies have revealed a diverse community, led by the phyla Bacteroidetes and Firmicutes.*

*• Although cockroaches may carry pathogenic bacteria, most strains are innocuous and may be useful for biotechnological applications.*

**Figure:**
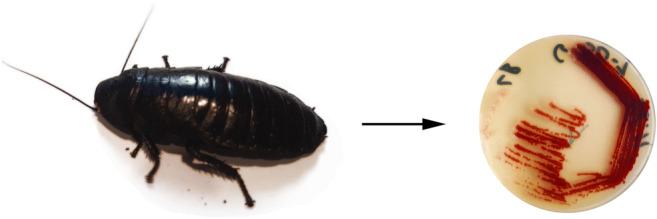
Graphical abstract

**Supplementary Information:**

The online version contains supplementary material available at 10.1007/s00253-020-10973-6.

## Introduction to cockroach biology and evolution

Cockroaches are hemimetabolous insects of the order *Blattodea*, which also includes termites. Their distinguishing morphological characters include a flattened body, a shield-like laminar structure (pronotum) covering the head and thorax, and the presence of tegmena (sclerotized forewings) and long antennae (Dettner and Peters 2010). Cockroaches are resilient insects, and most species can endure prolonged starvation and dehydration. For example, *Periplaneta americana* can survive for ~ 1 month without food or water (Willis and Lewis 1957). Cockroaches, like other insects, can become cannibalistic when nutrients are scarce, but cannibalism is not observed exclusively during starvation: some female cockroaches eat males after copulation in a similar manner to mantises (Richardson et al. 2010). Cockroaches can also reproduce by parthenogenesis, although fewer eggs are laid in comparison to sexual reproduction (Wharton and Wharton 1957). In addition, some female cockroaches can store sperm for life after a single copulation event, allowing eggs to be fertilized and laid without the presence of a male (Bell et al. 2007). Almost all cockroaches harbor endosymbiotic bacteria conferring the ability to recycle nitrogen from urea and ammonia wastes back to amino acids (Sabree et al. 2009). Overall, these biological mechanisms contribute to the fitness and success of cockroaches in the face of environmental challenges.

**Fig. 1:**
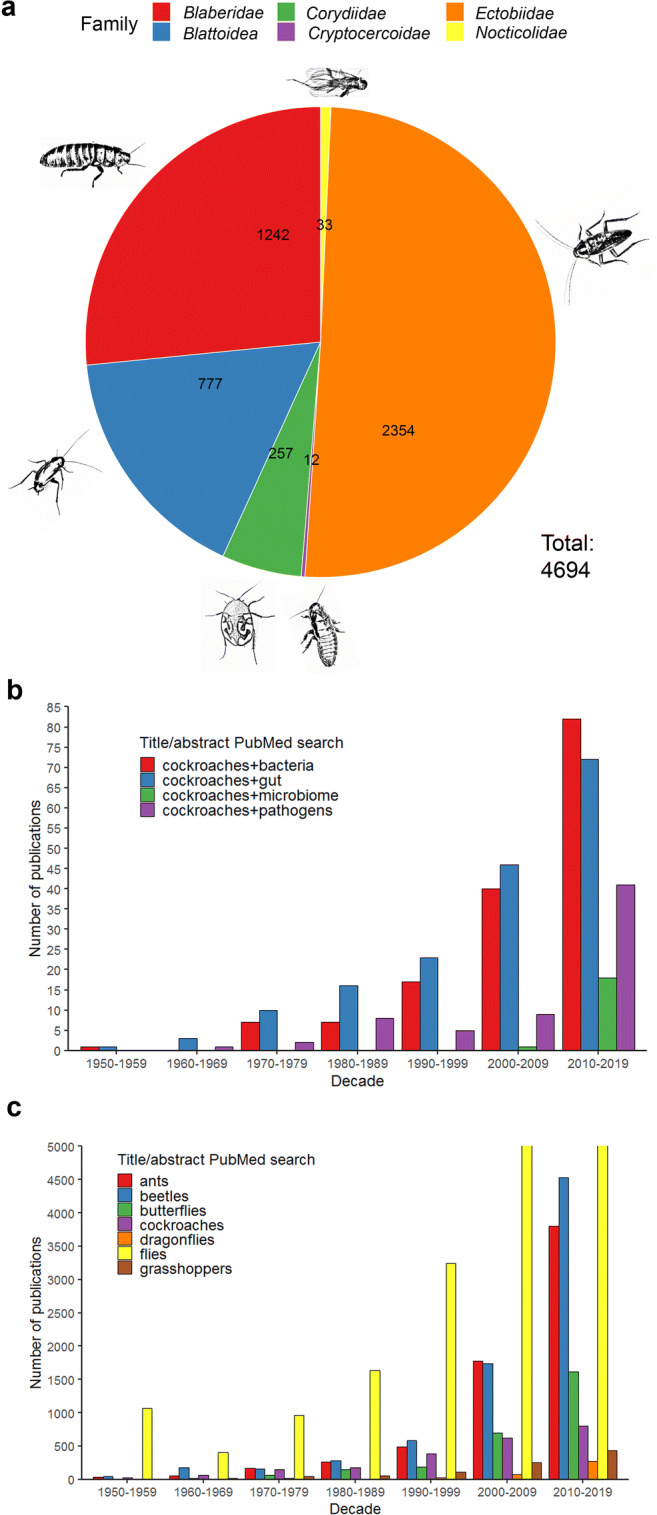
Frequency of research articles focusing on cockroaches and specific families thereof. **a** Number of cockroach species in each family of the order *Blattodea* (excluding termites). **b** Number of publications found in PubMed using the term cockroaches + (bacteria, gut, microbiome or pathogens) in the title, abstract, and keywords by decade since 1950. **c** Number of publications found in PubMed using search terms representing different orders of insects in the title, abstract and keywords by decade since 1950

The order *Blattodea* is divided into three superfamilies: *Blaberoidae*, *Blattoidea* (which also contains the termites), and *Corydiodea* (Wang et al. 2017; Ware et al. 2008). The fossil register shows a large number of cockroach-like (roachoid) specimens dating from the Late Carboniferous period spanning between 323 and 299 Mya (Zhang et al. 2013b). Such carboniferous fossils, which were abundant by 315 Mya, feature a long, external oviposition system (Hornig et al. 2018). They are considered to be ancestors of other taxa, including the mantises (*Mantodea*), and are thus not true cockroaches (Legendre et al. 2015). The greatest diversification leading to modern cockroaches occurred during the Cretaceous, probably in Siberia (Roth 2003; Vršanský 2004). Today (August 2020), there are 4685 species of cockroaches registered in the cockroach online database (Beccaloni 2014). The superfamily *Blaberoidea* is the largest with 3596 species, followed by *Blattoidea* with 799, and *Corydiodea* with 290 (Online Resource 1). The families with the greatest number of species are *Ectobiidae* and *Blaberidae* (both from superfamily *Blaberoidea*), with 2354 and 1242 species, respectively (Fig. 1a). The genus with the largest number of species is *Rhabdoblatta* with 151 species, followed by *Ischnoptera* with 100, *Phyllodromica* with 96, and *Balta* with 91. Surprisingly, there are only seven published articles that contain the word “*Rhabdoblatta*” in the title, and these are exclusively observational studies with species descriptions. We observed similar trends for most other cockroach genera. The exceptions were *Pe*. *americana* and *Blattella germanica*, which are common household cockroaches often considered as domiciliary pests. Most research on cockroach-associated microbes has focused on evidence that cockroaches carry pathogens (Rampal et al. 1983; Roth and Willis 1957; Strand and Brooks 1977). PubMed searches with “pathogens + cockroaches” in the title, abstract and keywords generated about half as many hits as “bacteria + cockroaches” and “gut + cockroaches,” and hits for the query “microbiome + cockroaches” only appeared in the last two decades, evidently because the term microbiome is relatively recent (Fig. 1b). Insect research has focused predominantly on flies, with more than 12,000 publications in the last decade, followed by beetles, ants, and butterflies (Fig. 1c). Cockroaches have received comparatively little attention, with ~ 1000 publications in the last decade, but still more than dragonflies and grasshoppers. Interest in cockroach research has increased due to their use as food/feed and as a source of pharmaceutical ingredients in China (Feng et al. 2018; Gao et al. 2018). More than 99% of cockroach species are not domiciliary pests, instead of living among decaying leaves, under tree bark, under stones, or in soil, and this large and untapped resource is likely to provide a bountiful source of microbial diversity. Several recent studies have suggested that the exploration of cockroach microbiomes will reveal as yet unclassified and uncultivated bacterial taxa (Lampert et al. 2019; Richards et al. 2017; Tegtmeier et al. 2018; Tinker and Ottesen, 2016). In this systematic review, we therefore discuss the collective body of literature on cockroach-associated bacteria identified either by conventional isolation and cultivation methods or by molecular biology. We assess whether these bacteria pose a danger as pathogens or constitute a reservoir of unexplored diversity with potential applications for biotechnology, medicine, agriculture, and industry.

## General outlook for cockroach-associated bacteria

Wild insects are associated with microbes under varied levels of dependence, ranging from mutualistic primary symbionts typically living inside specialized cells (bacteriocytes or mycetocytes) to facultative secondary symbionts often circulating in the gut and essentially integrating into a variable community (Moya et al. 2008). Cockroaches are an interesting model for studying bacterial endosymbiosis, because obligate intracellular *Blattabacterium* species are found stably in the order *Blattodea* but not in termites, they are transferred vertically, they have yet to be cultured outside their hosts, and they are involved in nitrogen recycling (Brooks and Richards 1966; Perru 2007; Sabree et al. 2009). Given their variable nature, cockroach secondary symbionts are less stable, but both culture-dependent and culture-independent studies have shown that some taxonomic clades are encountered more frequently. Cockroach gut bacteria are systematically transmitted by coprophagy because feces are often meals and constitute the first nutrient provided by filial proctodeal trophallaxis to neonates (Nalepa et al. 2001). Cannibalism, necrophagy, and feeding with exuviae (Bell et al. 2007) are alternative routes for the transmission of microflora across a colony. The three major anatomical compartments of the cockroach gut have marked differences in pH, redox potential, and hydrogen pressure (Cruden and Markovetz 1987; Lampert et al. 2019; Schauer et al. 2012; Vinokurov et al. 2007). The central portions of the cockroach midgut and the hindgut tend to be anoxic and therefore provide a good source of anaerobic bacteria.

Cockroaches are unable to synthesize certain amino acids and either acquire them in their diet or rely on symbionts. Blattabacteria are aerobic flavobacteria located within bacteriocytes in the cockroach fat tissue, but always in close proximity to specialized uric acid–containing urocytes (Brooks 1970). Detailed genomic analysis of *Blattabacterium* sp. BPLAN from *Pe*. *americana* revealed that 13% of its protein-encoding genes are required for amino acid biosynthesis and 7.8% are needed for the production of coenzymes (Sabree et al. 2009). Under deprivation, the insect host transforms uric acid (stored as urate in urocytes) into urea, which is then imported by blattabacteria and converted to ammonia (Patiño-Navarrete et al. 2014). The blattabacterial enzyme glutamate dehydrogenase (GdhA) catalyzes the addition of ammonia to 2-oxoglutarate yielding d-glutamic acid, which can be then transformed into most other amino acids, with the exception of l-asparagine and l-glutamine provided by the host (Patiño-Navarrete et al. 2014; Sabree et al. 2009). The blattabacterial pathways for the synthesis of glycine, l-methionine, l-proline, and l-serine are incomplete, but glycine and l-proline are abundant in the hemolymph, and thus, the maintenance of these pathways is unnecessary (Patiño-Navarrete et al. 2014). Some *Blattabacterium* strains (particularly those from xylophagous cockroaches) have fully or partially lost their ability to synthesize certain amino acids, but they all retain full pathways for l-alanine, l-aspartic acid, l-glutamic acid, l-histidine, l-phenylalanine, and l-tyrosine biosynthesis (Tokuda et al. 2013; Vicente et al. 2018). The knowledge gained from studying amino acid biosynthesis in *Blattabacterium* may be used in the future for the design of synthetic organisms that sustain the production of amino acids, for example, in waste-management settings.

The brain lysate and hemolymph of *Pe*. *americana* showed antibacterial activity against *Staphylococcus aureus* and *Escherichia coli* (Ali et al. 2017; Latifi et al. 2015). Because the antimicrobial activity of the hemolymph was induced by abdominal sublethal injection with viable *E*. *coli* cells (Basseri et al. 2016), innate immunity is probably involved in the response (Kim et al. 2016). Although cockroach antimicrobial peptides such as periplanetasin-2 are probably involved in the antimicrobial effect of the hemolymph (Lee et al. 2019; Yun et al. 2017), the activity is retained in ethanol and ethyl acetate extracts that would denature proteins (Kui et al. 2013). This suggests the presence of small antimicrobial molecules which may be of microbial origin (Ali et al. 2017). In addition, the feces of the xylophagous cockroach *Cryptocercus punctulatus* showed antifungal activity linked to compounds produced by gut microbes (Rosengaus et al. 2013). Very recently, a species of *Streptomyces* producing actinomycin X_2_ and collismycin was isolated from the gut of *Pe*. *americana* (Chen et al. 2020). Bacteria in cockroaches are likely to produce antimicrobial compounds less toxic than those found in soil bacteria, because the host must survive exposure to these agents. Insects have been recognized as important sources of microorganisms that produce bioactive molecules (Beemelmanns et al. 2016; Shi and Bode 2018), and cockroaches in particular, given their distinct evolutionary history and resilient nature, are home to bacteria with interesting metabolic capabilities (Table 1).

**Table 1:** Summary of bacterial strains isolated from cockroaches with potential biotechnological applications

Phylum	Species/strain	Sources	Biotechnological application	References
*Actinobacteria*	*Streptomyces globisporus* WA5-2-7	*Periplaneta americana*	Actinomycin X2 and collismycin A antibiotics	Chen et al. (2020)
*Firmicutes*	*Bacillus cereus* B1	*Blaberus craniifer*	Insecticidal phospholipase C	Ratcliffe and Rowley (1984); Rahmet-Alla and Rowley (1989); Rahmet-Alla and Rowley (1990)
	*Bacillus* sp. 29K	*Periplaneta americana*	Keratinolytic and proteolytic enzymes	Sharma et al. (2019)
	*Bacillus subtilis* BGI-1	*Blatte. germanica*	Fungicidal metabolites	Huang et al. (2013)
	*Enterococcus faecalis* E18	Undefined cockroach	Antibacterial bacteriocins	David et al. (2012)
	*Breznakia blatticola* ErySL	*Shelfordella lateralis*	Formate, ethanol, acetate producer	Tegtmeier et al. (2016a)
	*Clostridium* sp.	*Eublaberus posticus*	Carboxymethylcellulose decomposer	Cruden and Markovetz (1979)
*Proteobacteria*	*Shimwellia blattae* DSM 4481	*Blatta orientalis*	Cobalamin producer	Andres et al. (2004); Brzuszkiewicz et al. (2012); Burgess et al. (1973)
	*Pseudomonas reactans* BGI-14	*Blattella germanica*	Fungicidal metabolites	Zhang et al. (2013a)
	*Pseudomonas aeruginosa* BGf-2	*Blattella germanica*	Antifungal protein producer	Zhang et al. (2020)
	*Stenotrophomonas maltophilia* OG2	*Blatta orientalis*	Endosulfan degradation	Ozdal et al. (2017)
*Spirochaetes*	*Alkalispirochaeta cellulosivorans* JC227	*Cryptocercus punctulatus*	Cellulose degradation at basic pH	Sravanthi et al. (2016)
*Verrucomicrobia*	*Ereboglobus luteus* Ho45	*Shelfordella lateralis*	Anaerobic degradation	Tegtmeier et al. (2018); Wang et al. (2018)

## Are cockroaches truly responsible for human diseases?

Many cockroaches have been sampled in hospitals and health centers, and given the nature of the bacteria cultivated from these species, the insects have been blamed for nosocomial infections (Fakoorziba et al. 2014; Fotedar et al. 1991; Gliniewicz et al. 2003; Pai et al. 2004; Tilahun et al. 2012). Two classical studies linked the eradication of cockroaches in health centers to a lower frequency of infections, particularly with *Salmonella typhimurium* (Graffar and Mertens 1950), and to lower rates of hepatitis (Tarshis 1962). There is strong evidence that *Helicobacter pylori*, mycobacteria, *Pseudomonas aeruginosa*, *Salmonella* spp., and other bacteria can survive passage through the cockroach digestive system (Allen 1987; Ash and Greenberg 1980; Clot and Vago 1970; Fotedar and Banerjee 1993; Imamura et al. 2003; Klowden and Greenberg 1977; Stek 1982). Because these insects typically feed on decaying organic material, there is little doubt that they can disseminate pathogenic bacteria. In addition, some of the bacterial isolates from cockroaches show antibiotic resistance (Abdolmaleki et al. 2019; Bouamamaa et al. 2010; Islam et al. 2016; Menasria et al. 2015; Pai et al. 2005; Prado et al. 2006; Tilahun et al. 2012). Taken together, these data support the general view that cockroaches are dangerous pests in human domiciles and healthcare settings, acting as vectors for bacterial infections. However, there is no direct proof that cockroaches spread nosocomial infections, and the link between eradication and lower infection rates could be related to a more general improvement in cleanliness. Undisputed evidence would ideally require experiments following Koch’s postulates, such as the confirmation of identical serotypes in cockroaches and hospital patients. To the best of our knowledge, such studies have seldom been conducted. In one study, *Klebsiella pneumoniae* strains in insects and humans were indistinguishable, supporting the involvement of cockroaches in the dissemination of infections (Cotton et al. 2000). However, an analogous study on *P*. *aeruginosa* showed that the strains were different (Saitou et al. 2010). A recent review highlighting the association between cockroaches and infectious diseases (Donkor 2019) concluded that, although cockroaches increase risk and should not be tolerated in hospitals, there is not yet any definitive proof of their direct involvement in the transmission of infections to patients.

## Cultivation-dependent vs cultivation-independent analysis

**Fig. 2:**
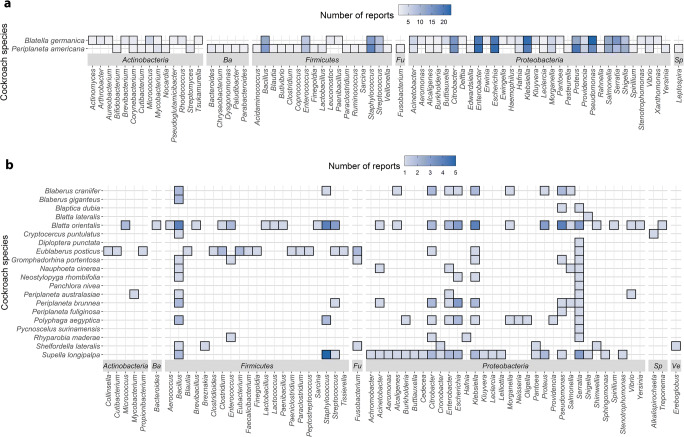
Genera of bacteria cultivated from cockroaches. **a** Cultivated bacteria isolated from the two most widely studied cockroaches: *Blatella germanica* and *Periplaneta americana*. **b** Cultivated bacteria isolated from lesser-studied cockroaches. The stronger the blue color, the higher number of reports discussing the cultivation of a bacterial species from a particular cockroach. *Ba*, *Fu*, *Sp*, and *Ve* refer to *Bacteroidetes*, *Fusobacteria*, *Spirochaetes*, and *Verrucomicrobia*, respectively. The absence of data (white boxes) does not imply the absence of a particular phylum, but rather the lack of corresponding reports

**Fig. 3:**
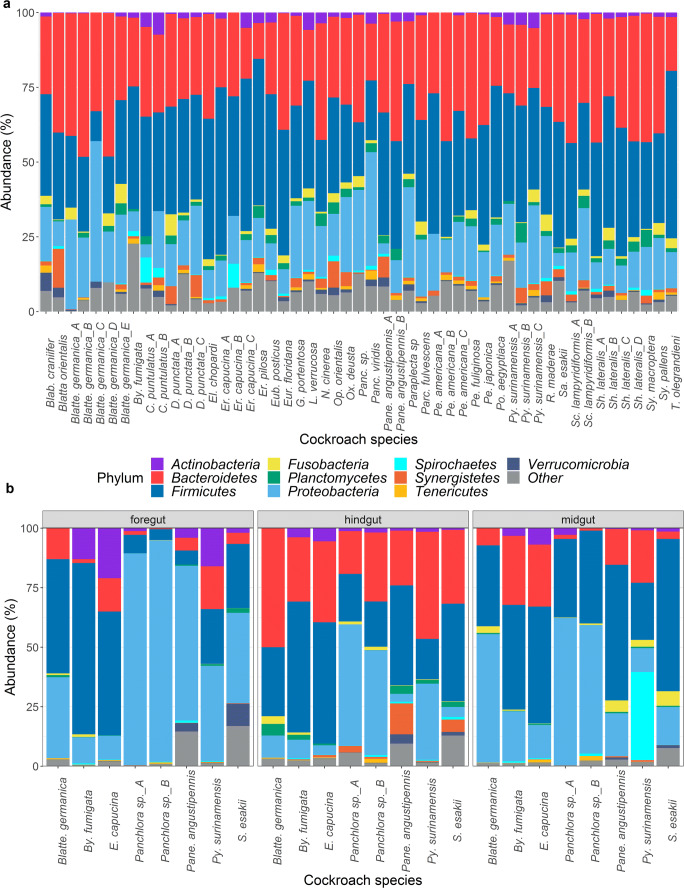
Relative abundance of bacterial phyla identified in cockroaches. **a** Comprehensive summary of literature studies reporting cockroach bacterial composition by phylum. Multiple studies involving the same species of cockroach are distinguished using capital letters (see Online Resource 3). **b** Differences in phyla composition (phylum level) in the foregut, midgut, and hindgut compartments (see Online Resource 4)

There is a large body of literature describing bacteria cultivated from cockroaches (Online Resource 2). To organize the data in a meaningful way, we generated a bidimensional heat map plot (Fig. 2). More than 20 studies have focused on the cultivation of bacteria from *Pe*. *americana* and *Blatte*. *germanica* (Akinjogunla et al. 2012; Cruden and Markovetz 1987; Fakoorziba et al. 2014; Oothuman et al. 1989; Rampal et al. 1983; Roth and Willis 1960; Strand and Brooks 1977; Wannigama et al. 2014). Other cockroaches including *Blatta orientalis* (Burgess et al. 1973) and *Eublaberus posticus* and *Supella longipalpa* (Le Guyader et al. 1989) have also been the subject of multiple cultivation studies (Cruden and Markovetz 1987; Roth and Willis 1960; Strand and Brooks 1977). Most bacteria cultivated from cockroaches represent the phylum *Proteobacteria* (Fig. 2). This may reflect the routine use of selective culture media in medical bacteriology (including MacConkey, deoxycholate citrate, and eosin methylene blue agars), which favor the recovery of Gram-negative bacteria. Further selectivity is introduced by the routine incubation temperature of 37 °C. Unsurprisingly, many studies have reported the cultivation of coliform bacteria (family *Enterobacteriaceae*, class *Gammaproteobacteria*) from cockroaches. A major advantage of the cultivation-dependent approach is that it facilitates the genomic, transcriptomic, proteomic, and metabolomic analyses of the isolated bacteria, allowing direct biotechnological exploitation. On the other hand, culture-independent methods offer a more authentic vision of the true diversity of a microbial niche and are considered the gold standard for the analysis of bacterial communities. However, the data should be interpreted with caution because it can be difficult to generalize due to the changing composition of communities over time and differences between individuals in the same community (Hornung et al. 2019; Schloss 2018). The results of microbiome studies are influenced by biological factors such as age, sex, and health; environmental factors such as diet, temperature, and humidity; and differences in analytical methods (DNA extraction, primers, sequencing, databases, and bioinformatics pipelines) although probably to a lesser extent. Higher termite gut microbiomes were among the first insect microbial communities to be studied because of the useful ability of the microorganisms to digest wood, explained by the activity of a consortium of bacteria and archaea (Brune and Dietrich 2015). Given their phylogenetic and ecological relationship with termites, cockroach microbiomes have been the subject of various studies and broad similarities in the structure of the communities have been reported (Online Resources 3 and 4). In contrast to cultivation approaches, the major phylum according to molecular studies is *Firmicutes*, followed by *Bacteroidetes* and finally *Proteobacteria*, together three accounting for more than 80% of the bacterial diversity (Fig. 3a).

### Firmicutes

*Firmicutes* is the second most frequently cultivated bacterial phylum from cockroaches (Fig. 2) but the most abundant phylum according to culture-independent methods, accounting for ~ 50% of the bacteria in some cockroaches such as *Ergaula* spp. (Dietrich et al. 2014; Tinker and Ottesen 2020). *Firmicutes* is most abundant in the midgut (~ 43%) compared to 30% in the foregut and 34% in the hindgut (Fig. 3b, Online resource 4). Given the more alkaline nature (pH 6.1–8.9) of the midgut (Vinokurov et al. 2007), a number of alkaliphilic, aerobic bacteria of the genera *Bacillus*, *Paenibacillus*, and *Enterococcus* flourish there (Yumoto et al. 2011).

The genus *Bacillus* has been readily cultivated from cockroaches, particularly strains related to *B*. *cereus* and *B*. *subtilis*. Molecular microbiome studies demonstrated the widespread presence in cockroaches of different clades of the family *Bacillaceae* including *Bacillus*, *Geobacillus*, *Oceanobacillus*, and *Ureibacillus* and also other related taxa such as *Paenibacillus* and *Lysinibacillus* and several unclassified clusters (Lampert et al. 2019; Mikaelyan et al. 2015; Schauer et al. 2014). A filamentous form of *B*. *cereus* known as Arthromitus attaches not only to gut epithelial cells in the cockroaches *Blaberus giganteum* and *Gromphadorhina portentosa* but also to termites, beetles, and some crustaceans (Margulis et al. 1998). The Arthromitus form is found in healthy cockroaches, and poor diet is associated with its depletion (Feinberg et al. 1999). *B*. *cereus* and related species such as *B*. *anthracis* and *B*. *thuringiensis* can cause disease in humans and other animals including insects because these bacteria can secrete lytic enzymes and toxins (Ehling-Schulz et al. 2019). The *B*. *cereus* strain B1 isolated from *Blaberus craniifer* (Ratcliffe and Rowley 1984) was shown to be highly pathogenic toward the cockroach *Rhyparobia maderae*, and a phospholipase C was found to be responsible for this effect (Rahmet-Alla and Rowley 1989; Rahmet-Alla and Rowley 1990). The *B*. *subtilis* strain BGI-1 isolated from *Blatte*. *germanica* showed strong fungicidal activity against *Beauveria bassiana*, but the active components were not identified (Huang et al. 2013). Another *Bacillus* strain cultured from *Pe*. *americana*, identified as the isolate 29K, showed the strongest keratinolytic and proteolytic activity among other *Bacillus* strains, achieving complete feather digestion in 7 days (Sharma et al. 2019). These examples demonstrate the biotechnological potential of *Bacillus* from cockroaches (Table 1), particularly the production of bioactive metabolites (Um et al. 2013) and industrially valuable enzymes, and also as a platform for the production of recombinant proteins (van Dijl and Hecker 2013).

The *Staphylococcus* genus has been isolated from six cockroaches, and *S*. *aureus* is the most frequently isolated species. According to culture-independent studies, staphylococci are abundant in the hindgut of *Shelfordella lateralis* (Schauer et al. 2014), but less abundant in other cockroaches (Lampert et al. 2019). Although *S*. *aureus* is a commensal bacterium that asymptomatically colonizes the skin and oral mucosa of ~ 30% of the human population, it can also gain virulence and cause dangerous infections (Gorwitz et al. 2008). The prominent presence of *S*. *aureus* in cockroaches has shaped the latter’s reputation as a disseminator of pathogens, particularly given the isolation of antibiotic-resistant *S*. *aureus* strains from some hospital cockroaches (Abdolmaleki et al. 2019; Islam et al. 2016; Menasria et al. 2014b). *S*. *aureus* has also been isolated from ants, butterflies, flies, gnats, moths, and wasps from two Brazilian hospitals (Oliveira et al. 2014).

The order *Lactobacillales* is prevalent in cockroaches, and particularly the genera *Enterococcus* and *Streptococcus* have been cultured from *Blatta orientalis*, *Blatte*. *germanica*, and *Pe*. *americana* (Online Resource 2). The genus *Lactobacillus* has been isolated from *Blatta orientalis* and *Pe*. *americana*, and both homofermentative and heterofermentative classes have been reported to proliferate in the gut (Kane and Breznak 1991). In culture-independent studies, *Lactobacillales* were particularly abundant in the crops of the leaf litter cockroaches *Ergaula capuchina* and *Byrsotria fumigata* accounting for more than 40% of the species (Lampert et al. 2019). Interestingly, ~ 50% of the reads from this group were assigned to unclassified taxa, including a group labeled “cockroach cluster”, suggesting that there exists a particular bacterial clade uniquely present in cockroaches that should be targeted for cultivation. Enterococci and lactobacilli are common gut bacteria (Walter 2008), and although their precise role is unclear, they probably improve the digestibility of food by transforming sugars into acetate and lactate, which are readily absorbed (Kane and Breznak 1991; Moreno et al. 2006). Some enterococci are opportunistic pathogens associated with nosocomial infections (Lebreton et al. 2014), and in insects, their translocation to the hemolymph is associated with disease (Mason et al. 2011). On the other hand, feeding insects with enterococci or lactobacilli can prevent death caused by *B*. *thuringiensis* (Grau et al. 2017) and other entomopathogens (Arredondo et al. 2018; Daisley et al. 2017; Rossoni et al. 2018). Enterococci are known to produce bacteriocins to suppress the growth of competing bacteria (Franz et al. 2007), and because they are naturally present in many artisanal food products, they can be used in the food industry either directly as a starter culture or as a source of purified antibiotic peptides (Khan et al. 2010). For example, nisin is currently an authorized food preservative (Younes et al. 2017) for sausage, cheese, and other meat and dairy products because it is a safe, thermostable during food processing but degradable by gastric and pancreatic proteases, thus having no effect on native gut microbes. The strain *Enterococcus faecalis* E18 isolated from a cockroach in Nigeria was shown to produce an enterocin or mixture of bacteriocins with strong antibacterial activity (David et al. 2012). Lactobacilli isolated from the honey bee have already been proposed as alternative probiotics (Parichehreh et al. 2018) and similar concepts could be applied to lactobacilli from cockroaches.

Anoxic portions of the cockroach midgut and hindgut are home to anaerobic bacteria and in particular members of the order *Clostridiales*, but specialized techniques are needed to cultivate them and only a few such studies have been reported in the literature. Species of the genus *Clostridium* have been cultivated from *Blatta orientalis* (Roth and Willis 1960) and *Eu*. *posticus* and *Pe*. *americana* (Cruden and Markovetz 1987). Other genera from the order *Clostridiales* cultivated from cockroaches (Online Resource 2) belong to the families *Clostridiaceae* (*Clostridium*, *Paeniclostridium*, *Paraclostridium*, *Sarcina*), *Eubacteriaceae* (*Eubacterium*), *Lachnospiraceae* (*Blautia*, *Butyrivibrio*, *Coprococcus*), *Oscillospiraceae* (*Faecalibacterium*, *Ruminococcus*), *Peptoniphilaceae* (*Finegoldia*), and *Peptostreptococcaceae* (*Clostridioides*, *Peptostreptococcus*) (Cruden and Markovetz 1987; Cruden and Markovetz 1979; Foglesong et al. 1984). Members of two less common *Firmicutes* classes (*Tissierellia* and *Erysipelotrichia*) have also been cultivated from cockroaches. The species *Tissierella preacuta* was cultivated from *Eu*. *posticus* (Cruden and Markovetz 1987) and *Breznakia blatticola* was isolated from *Sh*. *lateralis* (Tegtmeier et al. 2016a). *B*. *blatticola* is an anaerobic rod-like bacterium that produces formate, ethanol, and acetate from d-glucose. Moreover, the families *Lachnospiraceae* and *Ruminococcaceae* from the order *Clostridiales* are particularly well represented (5–28%) in the hindguts of all cockroaches (Lampert et al. 2019). In terms of abundance, three clusters are prominent: the gut cluster of *Lachnospiraceae*, and two clusters of *Ruminococcaceae*, one related to insects and one specific to cockroaches and termites, the latter being particularly abundant in omnivorous cockroaches (Lampert et al. 2019). The anaerobic family *Peptostreptococcaceae* is abundant in *Ergaula capucina* (3–6% of relative abundance), *Rhyparobia maderae*, *Ellipthorina chopardi*, and *Sh*. *lateralis* but not in other cockroaches (Lampert et al. 2019). Another moderately abundant clade of the order *Clostridiales* is the anaerobic or microaerophilic family *Veillonellaceae* (0.5–6% relative abundance in omnivorous cockroaches and *Panesthia angustipennis*). Interestingly, the *Veillonellaceae* family has been considered as a potential source of probiotic bacteria in animal husbandry because it reduces lactic acid accumulation in the rumen, which enhances overall productivity, but also prevents the colonization of swine and chicken by pathogenic bacteria (Marchandin and Jumas-Bilak 2014). The family *Christensenellaceae* was defined in 2012 based on a species recovered from human feces (Morotomi et al. 2012) and is an important focus of research today because it promotes health and is considered a probiotic (Waters and Ley 2019). Members of this family have been also detected by molecular methods in *Blatte*. *germanica* (Carrasco et al. 2014; Kakumanu et al. 2018; Zhang and Yang 2019) and *Diploptera punctata* (Ayayee et al. 2017,) but no species have yet been cultured from cockroaches. Some *Clostridium* strains isolated from cockroaches can degrade carboxymethyl cellulose (Cruden and Markovetz 1979) and could be used in biorefineries and biomass conversion processes to convert inexpensive and abundant cellulose into fermentable sugars, which can be then transformed into acetic acid, acetone, butanol, ethanol, or other commodity products (Cheng et al. 2019). Two *Clostridium* species, one isolated from termites and one from soil, have been used together to transform cellulose into the clean fuel hydrogen (Gomez-Flores et al. 2017). Diets rich in complex carbohydrates such as bran can enhance the production of hydrogen in the cockroach gut (Schauer et al. 2014). This is probably mediated by the abundant members of the order *Clostridiales*, perhaps with assistance from the phylum Bacteroidetes. Acetogenesis by CO_2_-reduction also occurs in cockroach guts and members of the *Clostridiales* Lowell cluster A (Lovell and Leaphart 2005) are partly responsible for this conversion (Ottesen and Leadbetter 2010). The capture of atmospheric CO_2_ using such processes could help to mitigate climate change.

### Bacteroidetes

Culture-independent studies indicate that the phylum *Bacteroidetes* is second after *Firmicutes* in terms of relative abundance in most cockroaches (Lampert et al. 2019), but in some species, such as house-dwelling cockroaches of the genera *Periplaneta* and *Blattella*, *Bacteroidetes* is often the most prominent phylum (Kakumanu et al. 2018; Rosas et al. 2018; Tinker and Ottesen, 2016). *Bacteroidetes* species preferably colonizes the cockroach hindgut (Fig. 3b, Online resource 4), which features anoxic and reducing conditions (Bauer et al. 2015; Lampert et al. 2019; Schauer et al. 2012). Despite their abundance, few cockroach-derived *Bacteroidetes* have been cultured (Cruden and Markovetz 1987; Dugas et al. 2001; Roth and Willis 1960; Vera-Ponce de León et al. 2020) (Fig. 2, Supplementary Table 2). The *Bacteroidetes* are non-sporulating obligate anaerobes that can metabolize complex polysaccharides such as cellulose, chitin, pectin, and xylan (Thomas et al. 2011). The order *Bacteroidales* is the most abundant in cockroaches, and the most prevalent families are the *Porphyromonadaceae* in cockroach that feed on leaf litter and wood, and *Rikenellaceae* in omnivorous cockroaches (Lampert et al. 2019). Within the family *Porphyromonadaceae*, two clades are prominent: one related to the genus *Dysgonomonas*, and the other is a yet unclassified group known as the “cockroach cluster.” Novel *Dysgonomonas* species have been isolated from termites (Pramono et al. 2015; Yang et al. 2014), and others have been recently cultured from *Pe*. *americana* (Vera-Ponce de León et al. 2020). From the family *Rikenellaceae*, the most important group is the genus *Alistipes*, which is particularly abundant in omnivorous cockroaches, but no individual species has been isolated from cockroaches thus far. The genus *Bacteroides* (order *Bacteroidales*) is present in the midgut and hindgut of cockroaches that feed on leaf litter but has not been found in wood-feeding cockroaches (Lampert et al. 2019), suggesting these bacteria digest complex polysaccharides such as cellulose, starch, and pectin, a finding corroborated by whole-genome sequencing and in vitro assays on cockroach-derived *Bacteroides*, *Dysgonomonas*, *Paludibacter*, and *Parabacteroides* isolates (Vera-Ponce de León et al. 2020). These bacteria produce powerful degrading enzymes with potential applications in the food/feed, textiles, paper, and cosmetics industries, as well as agriculture.

A yellow *Chryseobacterium* strain FR2 from the order *Flavobacteriales* was isolated from the hindgut of *Pe*. *americana* and cultured under anoxic conditions (Dugas et al. 2001). Given that *Blattabacterium* belongs to the order *Flavobacteriales*, extant cultivable bacteria representing this order in cockroaches are interesting because they could be intermediate forms from which *Blattabacterium* evolved. Moreover, *Chryseobacterium* species are useful in the context of industrial biotechnology because they produce flexirubin pigments (Siddaramappa et al. 2019) and hydrolytic enzymes (Brandelli and Riffel 2005; Gandhi et al. 2009).

### Proteobacteria

The *Proteobacteria* are the most frequently cultivated bacterial phylum from cockroaches, and members of the class *Gammaproteobacteria* are particularly abundant. *Proteobacteria* are most abundant in the foregut (~ 48%) and less so in the midgut (~ 31%) and hindgut (~ 19%) (Fig. 3b, Online resource 4), probably reflecting the more acidic and aerobic environment of the foregut (pH 5.0–6.8) which may be optimal for some clades of the phylum (Lampert et al. 2019). The order *Enterobacterales* (particularly the family *Enterobacteriaceae*) is the most abundant according to molecular studies (Lampert et al. 2019), followed by the orders *Xanthomonadales* and *Pseudomonadales*. This trend is supported by the frequency of cultivation, with the exception of *Xanthomonadales* which has been cultivated less often than *Pseudomonadales*. *Serratia* is the most frequently isolated genus from cockroaches (Fig. 2). There are 21 validated species of *Serratia* according to the List of Prokaryotic names with Standing in Nomenclature (LPSN, lpsn.dsmz.de) (Parte et al. 2020), and five of these (*Serratia liquefaciens*, *S*. *marcescens*, *S*. *odorifera*, *S*. *plymuthica*, and *S*. *rubidaea*) have been isolated (Elyasigomari et al. 2017; Menasria et al. 2014a; Pai et al. 2005; Roth and Willis 1960). However, identification in all cases was based on phenotypic and biochemical characters, which are not as precise as 16S rRNA gene sequencing. *Serratia* species have been found in healthy, sick, and dead insects, and they are residents of the normal insect community (Grimont and Grimont 2006; Grimont et al. 1979). They can also be opportunistic pathogens, and *S*. *marcescens* in particular often causes red disease in crowded colonies (Cruden and Markovetz 1979; Petersen and Tisa 2013). Chitinases and proteases secreted by *S*. *marcescens* are highly toxic in the insect hemocoel (Kaška et al. 1976; Lysenko 1976). *Serratia entomophila* is responsible for amber disease in the grass grub *Costelytra zealandica* mediated by the expression of plasmid-encoded Sep toxin, which inhibits the secretion of digestive enzymes (Gatehouse et al. 2009). Other *Serratia* species are clearly mutualistic, for example *Serratia symbiotica* is a cosymbiont of *Buchnera aphidicola* in aphids, providing essential metabolites to the host (Lamelas et al. 2011). *Serratia* species are known to produce specialized metabolites (Heise et al. 2019; Petersen and Tisa 2013), and given the abundance of cultured *Serratia* strains from cockroaches, a detailed investigation involving sequencing and the detection of biosynthetic gene clusters should be undertaken. Other members of the family *Enterobacteriaceae* and in particular the genera *Citrobacter*, *Enterobacter*, *Escherichia*, and *Klebsiella* have been frequently been isolated from cockroaches (Fig. 2), whereas other genera are less prominent. Although a number of these *Enterobacteriaceae* can be important human pathogens, there is a wide range of lifestyles and diversity in genotypes and phenotypes, and thus, pathogenicity cannot be inferred from taxonomy. The biotechnological applications of insect-derived *Enterobacteriaceae* include their ability to deliver toxic genes to pest insects (Zhang et al. 2010), their use as a source of plant-stimulating (Pan et al. 2019) and antimicrobial (Vivero et al. 2019) metabolites, and their use as in insect-rearing facilities (Augustinos et al. 2015; Azis et al. 2019). *Shimwellia blattae* (synonym: *Escherichia blattae*) was originally isolated from *Blatta orientalis* (Burgess et al. 1973) and later from other cockroaches (strains DSM 4481, ATCC 33429 and ATCC 33430), and interestingly, this species can synthesize cobalamin de novo and has been developed as a biotechnological source of vitamin B_12_ (Andres et al. 2004; Brzuszkiewicz et al. 2012).

The genera *Acinetobacter* and *Pseudomonas*, which belong to the order *Pseudomonadales*, have frequently been cultured from cockroaches (Fig. 2), and culture-independent studies have also revealed they are highly abundant (relative abundance > 16%) in the crop of *Pycnoscelus surinamensis* (Lampert et al. 2019). Human infections with *Acinetobacter baumanii* or *Pseudomonas aeruginosa* are difficult to treat in clinical practice due to the prevalence of antibiotic resistance, but no evidence of drug resistance was found in the *P*. *aeruginosa* strains isolated from cockroaches (Zarei et al. 2018). *P*. *aeruginosa* is the most frequently cultivated species of this genus, although cockroach-associated strains of *P*. *fluorescens*, *P*. *putida*, and *P*. *reactans* have also been reported (Online Resource 2). *Pseudomonas* species are very important in biotechnology, not only because they produce bioactive metabolites (Gross and Loper 2009) but also for their use in bioremediation (Wasi et al. 2013) and as a source of powerful lytic enzymes (proteases, lipases) for industrial processes. For example, the strains *P*. *reactans* BGI-14 (Zhang et al. 2013a) and *P*. *aeruginosa* BGf-2 (Zhang et al. 2020), isolated from *Blatte*. *germanica*, showed antifungal activity against the entomopathogenic fungus *Beauveria bassiana*. Furthermore, in culture-independent studies, the order *Xanthomonadales* was found to be abundant in the crops of xylophagous cockroaches *Pa*. *angustipennis* and *Salganea esakii* (Lampert et al. 2019), and members of this order (in particular strains of *Stenotrophomonas maltophilia*) have been cultured from *Blatta orientalis*, *Blatte*. *germanica*, and *Supella longipalpa* (Elgderi et al. 2006; Le Guyader et al. 1989; Mpuchane et al. 2006; Ozdal et al. 2016). The *S*. *maltophilia* OG2 isolated from *Blatta orientalis* was found to degrade the toxic organochlorinated pesticide endosulfan, utilizing it as a sulfur source, and transforming it to less toxic metabolites (Ozdal et al. 2017).

In addition to the *Gammaproteobacteria*, members of the *Alphaproteobacteria*, *Betaproteobacteria*, and to a lesser extent *Deltaproteobacteria* are relatively abundant in cockroaches based on cultivation-independent experiments (Lampert et al. 2019). *Alphaproteobacteria* are frequently identified (10–20% relative abundance) in the crops of cockroaches that feed on leaf litter and wood, particularly unclassified members of the order *Rhizobiales* and the family *Acetobacteraceae* (order *Rhodospirillales*). Both clades can fix atmospheric nitrogen, but this process has not been shown to occur directly in cockroaches, although hindgut protists can carry nitrogen-fixing bacteria (Tai et al. 2016). The *Acetobacteraceae* family is widespread in insects (Crotti et al. 2010) and although their role is unclear, members of this clade are useful for industrial oxidation reactions (such as the production of sorbose and acetic acid), so it is possible that cockroach-derived *Acetobacteraceae* could be used for this application in the future. A strain of *Sphingomonas paucimobilis* was isolated from *Su*. *longipalpa* (Le Guyader et al. 1989), and although this bacterium is an opportunistic human pathogen associated with nosocomial infections (Ryan and Adley 2010), it has also been applied in bioremediation (Coppotelli et al. 2008) and can synthesize gellan gum (Prajapati et al. 2013). Members of the class *Betaproteobacteria*, particularly those from the order *Burkholderiales* have also been cultured from cockroaches. The genera *Alcaligenes* (*Oligella*) and *Burkholderia* (Fig. 2, Online Resource 2) have been isolated from *Blatta orientalis*, *Blatte*. *germanica*, *Pe*. *americana*, *Polyphaga aegyptiaca*, and *Su*. *longipalpa* (Elyasigomari et al. 2017; García et al. 2012; Le Guyader et al. 1989; Roth and Willis 1960). *Burkholderia* species are important insect gut symbionts fulfilling diverse functions ranging from nutrition in stinkbugs to the protection of beetle eggs via the production of antifungal secondary metabolites (Kaltenpoth and Flórez 2020), but in cockroaches, their ecological function is unknown. The order *Burkholderiales* is the most abundant in cockroaches, particularly in the crop of *Py*. *surinamensis* and *Pa*. *angustipennis*, but the order *Rhodocyclales* is also frequently identified (18% relative abundance) in the gut of *Py*. *surinamensis* (Lampert et al. 2019). *Alcaligenes faecalis* lives within entomopathogenic nematodes and causes damage when injected into the hemocoel of *Galleria mellonella* (Quiroz-Castañeda et al. 2015). Insect-derived strains of *Alcaligenes* demonstrated antifungal activity (Shan et al. 2019) and together with the genus *Achromobacter*, isolated from *Su*. *longipalpa* (Le Guyader et al. 1989), they may produce bioactive specialized metabolites in a similar way to *Xenorhabdus* and *Photorhabdus*. Finally, *Deltaproteobacteria* are moderately abundant in the hindgut of omnivorous cockroaches, and particularly, the orders *Desulfobacterales* and *Desulfovibrionales* (Lampert et al. 2019) are useful for biotechnological processes because they reduce sulfate to sulfide (Trinkerl et al. 1990). They have been applied to contaminated soils, reducing the bioavailability of soluble toxic cadmium, which remains insolubly complexed with sulfide (Wang et al. 2019).

### Actinobacteria

*Actinobacteria* is an important phylum for biotechnology, because members of the family *Streptomycetaceae* produce a range of specialized metabolites. This phylum is generally abundant in cockroaches (13–21% relative abundance), although less so in species that feed on leaf litter feeding, and the orders *Bifidobacteriales* and *Corynebacteriales* are the most prevalent (Lampert et al. 2019). *Actinobacteria* are also found in the crop of xylophagous cockroaches but are much less abundant (1.7–4.1%). *Mycobacterium* is a frequently cultivated genus (Fig. 2, Online Resource 2), and because some species are human pathogens that survive passage through the cockroach digestive system (Fischer et al. 2003), cockroaches have been linked to the dissemination of mycobacterial disease (Allen 1987; Pai et al. 2003). The genera *Micrococcus*, *Corynebacterium*, and *Cutibacterium* are also frequently cultured from cockroaches (Cruden and Markovetz 1987). It is interesting to note that termites are a rich source of bacteria from the family *Streptomycetaceae* which perform functions that protect and maintain the colony (Kurtböke et al. 2015; Sujada et al. 2014), but few species have been isolated from cockroaches. The first reported cockroach-associated *Streptomyces* species was found to be carried by nematodes that recurrently infect cockroaches (Hoffman 1953), but the species “*Streptomyces leidnematis*” was not validated and it is currently not found in any collection. The *Streptomyces globisporus* strain WA5-2-7 was recently cultivated from the gut of *Pe*. *americana* and was found to match the insect clade of *S*. *albidoflavus* (Cheng et al. 2015; Matarrita-Carranza et al. 2017). The antibiotics actinomycin X2 and collismycin A, both with activity against methicillin-resistant *S*. *aureus* (MRSA), were purified from 40 L of *S*. *globisporus* WA5-2-7 culture broth (Chen et al. 2020), highlighting the biotechnological potential of cockroach-associated *Streptomyces*.

### Fusobacteria

The phylum *Fusobacteria* is found mostly in the midgut of wood-feeding cockroaches, with a 2–7% relative abundance, and in the hindgut of omnivorous cockroaches, with 0.2–6.3% relative abundance (Lampert et al. 2019). Strains belonging to the genus *Fusobacterium* have been cultured from *Eu*. *posticus*, *G*. *portentosa*, *Pe*. *americana*, and *Sh*. *lateralis* (Cruden and Markovetz 1987; Robertson 2007; Tegtmeier et al. 2016b). *Fusobacteria* are similar to *Bacteroidetes* (they are Gram-negative, non-sporulating, anaerobic bacteria) but they cluster in a different group based on 16S rRNA sequences (Staley and Whitman 2010). They are normal residents of the human oral cavity and the gut, but some species are pathogenic, for example, *Fusobacterium necrophorum* causes an oropharyngeal infection known as Lemierre’s syndrome (Riordan 2007), and *F*. *nucleatum* has been linked to colon cancer (Kostic et al. 2013). *F*. *necrophorum* has been isolated from *Eu. posticus* (Cruden and Markovetz 1987) and this cockroach is also home to the pleomorphic species *F. varium*, which switches between rod and cocci forms during its life cycle (Foglesong et al. 1984). Fusobacteria are used in biotechnological processes for the production of succinate (McDonald and White 2019) and also as a source of interesting enzymes for bioconversion applications (Silva et al. 2019; Tang et al. 2018).

### Spirochaetes

Spiral-shaped and in general anaerobic bacteria of the phylum *Spirochaetes* have been isolated from a few cockroaches, and specifically, the genera *Leptospira* (Gonzalez-Astudillo et al. 2015) and *Treponema* (Roth and Willis 1960) are relevant because some species from these genera are recognized human pathogens. Molecular studies have revealed that *Spirochaetes* are abundant in the hindgut of *C. punctulatus* (Dietrich et al. 2014) and *E. capucina* (Mikaelyan et al. 2015), reaching ~ 8% of relative abundance and also in the midgut of *Py. surinamensis* (Lampert et al. 2019). A new genus (*Alkalispirochaeta*) was proposed following the isolation of the strain JC227 from the wood-eating cockroach *C. punctulatus*, and other alkaliphilic spirochetes were added to the genus, including one species isolated from termites (Sravanthi et al. 2016). *Alkalispirochaeta cellulosivorans* JC227 can degrade cellulose at high pH and in presence of high salt concentrations and could therefore be suitable for the bioconversion of cellulosic materials in the detergent industry (Thapa et al. 2020).

### Planctomycetes

The phylum *Planctomycetes* is present in the hindgut of wood-feeding and omnivorous cockroaches with a relative abundance of 0.2–8.4% based on molecular studies (Lampert et al. 2019; Richards et al. 2017). Three clusters (one gut cluster and two “termite-cockroach”) of the family *Planctomycetaceae* are the most frequently detected in cockroaches, but they have not been validly described because a cultivable strain has yet to be isolated. *Planctomycetes* produce diverse specialized metabolites (Graça et al. 2016), and the discovery of new species with unknown metabolic potential could lead to new applications in pharmaceutical and agrochemical industries.

### Verrucomicrobia

Only a single bacterial strain from the phylum *Verrucomicrobia* has been cultivated from cockroaches, although the phylum is represented in wood-feeding and omnivorous cockroaches based on culture-independent studies (Lampert et al. 2019; Pietri et al. 2018). The microaerophilic species *Ereboglobus luteus* was isolated from the hindgut of *Sh. lateralis* (Tegtmeier et al. 2018). This bacterium can metabolize pectin, but is unable to develop in an aerobic environment. Interestingly, like almost all *Verrucomicrobia* isolated from insect guts, it belongs to the family *Opitutaceae*. *Verrucomicrobia* species are a major component of the degradation community in anaerobic sludge (Wang et al. 2018) and could therefore be developed for utilization in anaerobic biorefineries.

### Other phyla

Other phyla are less frequently found in cockroaches, although unique clades appear to exist in some species. For example, the family *Entomoplasmataceae* (phylum *Tenericutes*) reaches 16% relative abundance in the crop of *Sa. esakii* (Lampert et al. 2019), although no cockroach-derived species from this phylum have been cultivated. Similarly, the Candidatus *Tammella* genus of the phylum *Synergistetes* is abundant in the hindguts of *Blatta orientalis* and *Pa. angustipennis*, although the LPSN considers this clade as part of the phylum *Bacteroidetes* (Parte et al. 2020), reflecting the difficulty of valid assignments in the absence of cultured strains. The genera *Edaphobacter*, *Telmatobacter*, and other less-studied clades from the phylum *Acidobacteria* are relatively abundant (0.2–6%) in the crop of wood-feeding cockroaches (Lampert et al. 2019). Moreover, some uncultivated members of the small phyla *Lentisphaerae*, *Fibrobacteres*, and *Elusimicrobia* and the candidate phylum TM7 appear to be unique to cockroaches and termites (Lampert et al. 2019; Mikaelyan et al. 2015) and should be studied in detail not only for taxonomic description but also to assess their ecological role and their potential utilization in biotechnology.

## Conclusions

Most cockroach-associated bacteria belong to the phyla *Proteobacteria*, *Firmicutes*, and *Bacteroidetes*, together accounting for more than 80% of the total microbiome. The remaining 20% comprises less-abundant phyla such as *Actinobacteria*, *Fusobacteria*, *Planctomycetes*, *Verrucomicrobia*, and *Spirochaetes*. Among the readily cultivable bacteria from cockroaches, most studies have focused on the isolation of Gram-negative coliforms, staphylococci, and mycobacteria, and as a consequence, cockroaches have been proclaimed as unsanitary pests that pose a risk to health. Indeed, cockroaches can disseminate pathogens in their feces and should be eradicated from food preparation areas and healthcare settings. However, we consider cockroaches as a promising source of biotechnologically useful microorganisms, because they have co-evolved with bacteria, which are therefore likely to produce less toxic antimicrobials compared to soil bacteria in order to ensure the host survives. Cockroach-derived bacteria also appear to be easier to cultivate than bacteria associated with termites. Recent research has revealed the presence of less-explored taxa that could improve our understanding of microbe-host relationships and perhaps more importantly expand the biotechnological applications of microorganisms in biorefineries, bioremediation, and the development of pharmaceutical and agrochemical products and industrial enzymes.

## Electronic supplementary material

ESM 1 (PDF 555 kb)

## Data Availability

Not applicable.
