# Supplementary material for: Bacteria associated with cockroaches: health risk or biotechnological opportunity?
Source: Appl Microbiol Biotechnol. 2020 Oct 31;104(24):10369–87. doi: 10.1007/s00253-020-10973-6 (PMC7671988; doi:10.1007/s00253-020-10973-6)
Supplement: Supplementary file 1 — (PDF 555 kb) [file 253_2020_10973_MOESM1_ESM.pdf]

Journal of Applied Microbiology and Biotechnology

Mini-review

Bacteria associated with cockroaches: health risk or biotechnological opportunity?

Juan Guzman[1,*], Andreas Vilcinskas[1,2]

[1] Department of Bioresources, Fraunhofer Institute for Molecular Biology and Applied Ecology, Ohlebergsweg 12, D-35392 Giessen, Germany

[2] Institute for Insect Biotechnology, Justus-Liebig-University of Giessen, Heinrich-Buff-Ring 26-32, D-35392, Giessen, Germany

Corresponding author:

Juan Guzman

juan.guzman@ime.fraunhofer.de

Telefon +49 641 9937761

Fax +49 641 4808581

Online Resource 1. List of extant cockroach genera.

Superfamily	Family	Subfamily	Genera (number of species)
Blaberoidea (3596)	*Blaberidae* (1242)	*Blaberinae*	*Archimandrita* (2), *Aspiduchus* (4), *Bionoblatta* (4), *Blaberus* (19), *Blaptica* (17), *Byrsotria* (3), *Eublaberus* (9), *Hemiblabera* (7), *Hiereoblatta* (1), *Hormetica* (15), *Hyporichnoda* (6), *Lucihormetica* (11), *Minablatta* (3), *Monachoda* (4), *Monastria* (4), *Neorhicnoda* (1), *Oxycercus* (1), *Paradicta* (2), *Parahormetica* (5), *Petasodes* (3), *Phoetalia* (2), *Sibylloblatta* (2), *Styphon* (1)
		Diplopterinae	*Diploptera* (9)
		Epilamprinae	*Africalolampra* (1), *Alphelixia* (1), *Anisolampra* (1), *Antioquita* (2), *Aptera* (2), *Apsidopis* (2), *Ataxigamia* (2), *Audreia* (7), *Blepharodera* (2), *Calolampra* (28), *Calolamprodes (8)*, *Capucinella* (2), *Cariacasia* (1), *Colapteroblatta* (15), *Comptolampra* (1), *Cyrtonotula* (4), *Decoralampra* (1), *Dryadoblatta* (2), *Epilampra* (70), *Galilatta* (2), *Gurneya* (1), *Haanina* (11), *Homalopteryx* (2), *Howintoniella* (1), *Indoapterolampra* (1), *Litopeltis* (12), *Miroblatta* (2), *Molytria* (3), *Morphna* (12), *Notolampra* (3), *Opisthoplatia* (2), *Orchidoeca* (1), *Paracolamprodes* (1), *Phlebonotus* (2), *Phoraspis* (14), *Pinaconota* (4), *Placoblatta* (1), *Principola* (2), *Poeciloderrhis* (16), *Pseudophoraspis* (18), *Stictolampra* (18), *Rhabdoblatta* (151), *Rhabdoblattella* (4), *Rhicnoda* (4), *Thorax* (1), *Ylangella* (1)
		Geoscapheinae	*Geoscapheus* (6), *Macropanesthia* (14), *Neogeoscapheus* (4), *Parapanesthia* (2)
		Gyninae	*Alloblatta* (5), *Gyna* (31), *Paraprincisaria* (1), *Princisaria* (3), *Pseudocalolampra* (3)
		Oxyhaloinae	*Aeluroblatta* (1), *Ateloblatta* (3), *Brachynauphoeta* (6), *Coleoblatta* (1), *Elliptorrhina* (10), *Griffiniella* (4), *Gromphadorhina* (4), *Heminauphoeta* (2), *Henschoutedenia* (12), *Jagrehnia* (11), *Leozehntnera* (1), *Nauphoeta* (1), *Oxyhaloa* (10), *Princisa* (1), *Pronauphoeta* (4), *Rhyparobia* (7), *Simandoa* (1)
		Panchlorinae	*Achroblatta* (1), *Anchoblatta* (1), *Biolleya* (1), *Panchlora* (49), *Pelloblatta* (2)
		Panesthiinae	*Ancaudellia* (20), *Annamoblatta* (1), *Caeparia* (5), *Microdina* (1), *Miopanesthia* (8), *Panesthia* (59), *Salganea* (47)
		Paranauphoetinae	*Paranauphoeta* (22)
		Perisphaerinae	*Bantua* (6), *Compsagis* (3), *Corydidarum* (21), *Cyrtotria* (22), *Derocalymma* (16), *Ellipsica* (6), *Elliptoblatta* (13), *Glomerexis* (1), *Gymnonyx* (2), *Hostilia* (3), *Hyposphaeria* (24), *Laxta* (13), *Neolaxta* (3), *Perisphaerus* (17), *Pilema* (13), *Platysilpha* (3), *Poecicloblatta* (1), *Pseudoglomeris* (3), *Zuluia* (5)
		Pycnoscelinae	*Proscratea* (3), *Pynoscelus* (15), *Stilpnoblatta* (3)
		Zetoborinae	*Alvarengaia* (1), *Capucina* (1), *Lanxoblatta* (7), *Parasphaeria* (5), *Phorioeca* (7), *Phortioecoides* (1), *Schistopeltis* (3), *Schizopilia* (2), *Schultesia* (2), *Thanatophyllum* (1), *Tribonium* (14), *Tribonoidea* (1), *Zetobora* (6), *Zetoborella* (1)
		-	*Apotrogia* (3), *Cacoblatta* (1), *Cerocardia* (1), *Diplopterina* (2), *Elfridaia* (1), *Eustegasta* (16), *Evea* (1), *Glyptopeltis* (2), *Gynopeltis* (3), *Hedaia* (1), *Isoniscus* (3), *Kemneria* (1), *Mesoblaberus* (1), *Mioblatta* (1), *Paraplecta* (5), *Phenacisma* (2), *Progonogamia* (1), *Pseudogyna* (1), *Pseudoplatia* (1), *Stenoblatta* (1), *Stictomorphna* (2), *Thliptoblatta* (1), *Thoracopygia* (1)
	Ectobiidae (2354)	*Blattellinae*	*Anallacta* (12), *Anaplectella* (18), *Anapleictoidea* (13), *Aseucina* (1), *Asiablatta* (1), *Astylella* (2), *Attaphila* (6), *Beybienkoa* (22), *Blattella* (52), *Burchellia* (5), *Caboverdea* (2), *Caffroblatta* (2), *Cahita* (9), *Calhypnorna* (5), *Carbrunneria* (18), *Chorisia* (1), *Chrastoblatta* (2), *Chromatonotus* (14), *Dasyblatta* (8), *Dethieridris* (3), *Dewittea* (2), *Dyakinodes* (7), *Eowilsonia* (3), *Episymploce* (72), *Escala* (12), *Euandroblatta* (19), *Eudromiella* (9), *Euhypnorna* (1), *Haplosymploce* (11), *Hemithyrsocera* (69), *Hensaussurea* (11), *Hololeptoblatta* (2), *Hoplophoropyga* (8), *Hypnorna* (2), *Ignabolivaria* (2), *Ischnoptera* (100), *Jacobsonina* (13), *Johnrehnia* (36), *Keyella* (2), *Litoblatta* (11), *Loboptera* (32), *Lobopterella* (3), *Lobopteromorpha* (5), *Malaccina* (10), *Mayottella* (1), *Miriamrothschildia* (6), *Moluchia* (6), *Nelipophygus* (4), *Neoloboptera* (6), *Neoloboptermorpha* (1), *Neotemnopteryx* (14), *Neotrogloblattella* (1), *Nesomylacris* (7), *Nondewittea* (1), *Ornatiblatta* (1), *Parasigmoidella* (38), *Paratemnopteryx* (13), *Parcoblatta* (12), *Parectoneura* (1), *Phymatosilpha* (1), *Pseudoanaplectinia* (1), *Pseudoceratinoptera* (2), *Pseudomops* (44), *Pseudosigmella* (2), *Pseudothyrsocera* (13), *Robshelfordia* (13), *Saltoblattella* (1), *Scalida* (5), *Sigmella* (23), *Stayella* (8), *Symploce* (64), *Symplocodes* (8), *Tartaroblatta* (5), *Temnopteryx* (7), *Termitoblatta* (3), *Trogloblattella* (1), *Xestoblatta* (44), *Xosablatta* (13)
		Ectobiinae	*Arbiblatta* (9), *Capraiellus* (3), *Choristima* (12), *Ectobius* (68), *Ectoneura* (21), *Luridiblatta* (3), *Phyllodromica* (97), *Planuncus* (13), *Pseudectoneura* (1), *Stenectoneura* (5), *Theganopteryx* (31)
		Nyctiborinae	*Eunyctibora* (5), *Eushelfordia* (2), *Eushelfordiella* (1), *Megaloblatta* (4), *Muzoa* (3), *Nyctantonina* (2), *Nyctibora* (30), *Paramuzoa* (4), *Paratropes* (13), *Pseudischnoptera* (3)
		Pseudophyllondro miinae	*Afrobalta* (2), *Afroneura* (1), *Aglaopteryx* (11), *Agmoblatta* (1), *Allacta* (41), *Amazonina* (18), *Apteroblatta* (5), *Arawakina* (1), *Asemoblattana* (2), *Balta* (91), *Cariblatta* (77), *Cariblattoides* (13), *Ceratinoptera* (16), *Chorisoblatta* (16), *Chorisomaculata* (1), *Chorisoneura* (92), *Chorisoneurodes* (2), *Chorisoserrata* (3), *Delosia* (2), *Dendroblatta* (16), *Desmosia* (1), *Doradoblatta* (1), *Ellipsidion* (18), *Epibalta* (1), *Euphyllodromia* (44), *Euthlastoblatta* (9), *Helgaia* (17), *Hypnornoides* (2), *Imblattella* (13), *Isoldaia* (1), *Lanta* (3), *Latiblattella* (18), *Leuropeltis* (6), *Lophoblatta* (13), *Lupparia* (15), *Macrophyllodromia* (12), *Margattea* (48), *Margatteoidea* (6), *Matabelina* (13), *Mediastinia* (3), *Megamareta* (9), *Nahublattella* (17), *Neoblattella* (39),

			Pachnepteryx (4), Paranocticola (2), Phidon (4), Plectoptera (25), Prosoplecta (20), Pseudectobia (1), Pseudobalta (3), Pseudophyllodromia (8), Pseudosymploce (4), Rhytidometopum (3), Riatia (21), Shelfordina (26), Sliferia (4), Sorineuchora (11), Squamoptera (3), Sundablatta (4), Supella (10), Supellina (3), Tagaloblatta (1), Tomeisneria (2), Trioblattella (7)
		-	Africablatta (1), Akaniblatta (1), Alsteinia (4), Anareolaria (1), Aneurinita (3), Anisopygia (4), Antitheton (1), Aphlebiella (1), Aruistra (1), Astylloblatta (1), Atticola (1), Blattellina (1), Caloblatta (4), Celeriblattina (2), Ceuthobia (3), Ceuthobiella (2), Dictyoblattella (1), Dipteretrum (13), Discalida (1), Distichopis (1), Dysimploce (1), Drabeha (1), Duryodana (1), Dyakina (2), Eublattella (1), Euhebardula (4), Eulissosoma (1), Euloboptera (5), Eurylestes (1), Eutheganopteryx (1), Hanitschella (1), Hanitschia (1), Hanstroemidium (2), Hemipterisca (2), Hemipterota (1), Incoblatta (1), Liosilpha (2), Lobodromia (2), Lophometopum (3), Malloblatta (4), Meratina (3), Maretiola (1), Margattina (1), Microblatta (1), Myrmeblattina (1), Namablatta (2), Neoleptoblatta (1), Nimbablatta (1), Nisibis (1), Nymphodromia (1), Onycholobus (6), Operculea (1), Paraloboptera (1), Parascalida (1), Parellipsidion (3), Phorticolea (2), Piroblatta (3), Rudebeckia (3), Sciablatta (6), Simblerastes (1), Sinablatta (1), Sutteriana (1), Tairella (1), Xosaia (3)
Blattoidea (799)	Blattoidae (777)	Anaplectidae	Anaplecta (94), Maraca (1)
		Blattidae	Afrostylopyga (1), Anamesia (11), Angustonicus (10), Apterisca (1), Archiblatta (3), Blatta (14), Brinckella (1), Cartoblatta (14), Catara (2), Celatoblatta (25), Cosmozosteria (14), Deropeltis (51), Desmozosteria (11), Dorylaea (25), Drymaplaneta (6), Duchailluia (11), Eppertia (4), Eroblatta (1), Eumethana (1), Eurycotis (59), Euzosteria (10), Hebardina (17), Henicotyle (1), Homalosilpha (12), Leptozosteria (7), Macrocerca (11), Macrostylopyga (3), Maoriblatta (6), Megazosteria (5), Melanozosteria (44), Methana (11), Mimosilpha (1), Miostylopyga (1), Neostylopyga (28), Pallidionicus (5), Pelmatosilpha (24), Pellucidonicus (2), Periplaneta (53), Platyzosteria (62), Polyzosteria (17), Protagonista (1), Pseudoderopeltis (45), Pseudolampra (4), Punctulonicus (2), Rothisilpha (8), Scabina (1), Scabinopsis (1), Shelfordella (3), Temnelytra (3), Thyrsocera (3), Zonioploca (9)
		Lamproblattidae	Eurycanthablatta (1), Lamproblatta (8), Lamproglandifera (1)
		Tryonicidae	Lauraesilpha (11), Tryonicus (6)
	Cryptocercoidae (12)	Cryptocercidae	Cryptocercus (12)
	Termitoidae		Termites are not considered cockroaches and are not included in this review.
Corydiodea (299)	Corydiidae (257)	Corydiinae	Anisogamia (1), Arenivaga (48), Austropolyphaga (2), Eremoblatta (2), Ergaula (7), Eucorydia (14), Eupolyphaga (7), Hemelytroblatta (28), Heterogamisca (9), Heterogamodes (2), Homoeogamia (2), Hypercompsa (5), Leiopteroblatta (1), Mononychoblatta (1), Nymphrytria (1), Polyphagina (1), Polyphagoides (1), Therea (8)
		Euthyrrhaphinae	Euthyrrhapha (9)
		Holocompsinae	Holocompsa (10)
		Latindiinae	Buboblatta (2), Latindia (9), Sinolatindia (1)
		Tiviinae	Sphecophila (1), Tivia (15)
		-	Anacompsa (4), Bucolion (1), Compsodes (4), Ctenoneura (27), Homopteroidea (8), Ipisoma (1), Ipolatta (1), Melestora (8), Melyroidea (2), Myrmecoblatta (3), Oulopteryx (2), Paralatindia (4), Pholadoblatta (1), Zetha (4)
	Nocticolidae (33)	Nocticolidae	Alluaudellina (2), Cardacopsis (1), Cardacus (1), Metanocticola (1), Nocticola (21), Pholeosilpha (1), Spelaeoblatta (4), Typhloblatta (1), Typhloblattodes (1)

Online Resource 2. List of bacteria cultivated from cockroaches.

Cockroach	Bacteria			References
**Scientific name (family)**	**Phylum**	**Family**	**Scientific name**	
Blaberus craniifer (Blaberidae)	*Firmicutes*	*Bacillaceae*	*Bacillus cereus, B. subtilis*	(Ratcliffe and Rowley 1984; Roth and Willis 1960)
		Staphylococcaceae	*Staphylococcus aureus, S. epidermidis*	(Roth and Willis 1960)
	Proteobacteria	*Alcaligenaceae*	*Alcaligenes faecalis*	(Roth and Willis 1960)
		Enterobacteriaceae	*Citrobacter freundii*	(Roth and Willis 1960; Strand and Brooks 1977)
			Enterobacter cloacae	(Strand and Brooks 1977)
			Escherichia coli	(Strand and Brooks 1977)
			Klebsiella aerogenes	(Roth and Willis 1960; Strand and Brooks 1977)
			Morganella morganii	(Strand and Brooks 1977)
			Proteus vulgaris	(Roth and Willis 1960)
			Salmonella enterica, S. typhimurium	(Strand and Brooks 1977)
		Pseudomonaceae	*Pseudomonas aeruginosa*	(Roth and Willis 1960; Strand and Brooks 1977)
Blaberus giganteus (Blaberidae)	*Firmicutes*	*Bacillaceae*	*Bacillus cereus*	(Feinberg et al. 1999; Margulis et al. 1998)
Blaptica dubia (Blaberidae)	*Proteobacteria*	*Enterobacteriaceae*	*Serratia marcescens*	(Strand and Brooks 1977)
		Pseudomonadaceae	*Pseudomonas aeruginosa*	(Strand and Brooks 1977)
Blatta lateralis (Blattidae)	*Proteobacteria*	*Enterobacteriaceae*	*Shigella flexneri*	(Roth and Willis 1960)
Blatta orientalis (Blattidae)	*Actinobacteria*	*Micrococcaceae*	*Micrococcus luteus*	(Burgess et al. 1973; Roth and Willis 1960)
	Bacteroidetes	*Bacteroidaceae*	*Bacteroides fragilis*	(Roth and Willis 1960)
	Firmicutes	*Aerococcaceae*	*Aerococcus viridans*	(Burgess et al. 1973)
		Bacillaceae	*Bacillus atrophaeus, B. cereus, B. circulans, B. coagulans, B. firmus, B. licheniformis, B. megaterium, B. subtilis, B. thuringiensis*	(Burgess et al. 1973; Karimi Zarchi and Vatani 2009; Ozdal et al. 2016; Roth and Willis 1960; Strand and Brooks 1977)
		Clostridiaceae	*Clostridium cochlearium, C. sporogenes, C. ventriculi*	(Roth and Willis 1960)
			Sarcina sp.	(Roth and Willis 1960)
		Enterococcaceae	*Enterococcus durans, E. faecalis*	(Burgess et al. 1973; Roth and Willis 1960)
		Lactobacillaceae	*Lactobacillus fermentum*	(Roth and Willis 1960)
		Paenibacillaceae	*Brevibacillus brevis*	(Burgess et al. 1973)
			Paenibacillus larvae, P. polymyxa	(Burgess et al. 1973)
		Streptococcaceae	*Lactococcus lactis*	(Burgess et al. 1973)
			Streptococcus equinus, S. sanguinis, S. pyogenes	(Burgess et al. 1973; Karimi Zarchi and Vatani 2009; Roth and Willis 1960)
		Staphylococcaceae	*Staphylococcus aureus, S. epidermidis*	(Burgess et al. 1973; Karimi Zarchi and Vatani 2009; Menasria et al. 2015; Roth and Willis 1960)
	Proteobacteria	*Alcaligenaceae*	*Alcaligenes faecalis*	(Roth and Willis 1960)

		Enterobacteriaceae	*Citrobacter amolonaticus, C. diversus, C. freundii*	(Burgess et al. 1973; Karimi Zarchi and Vatani 2009; Ozdal et al. 2016)
			Enterobacter cloacae, E. sakazakii	(Burgess et al. 1973; García et al. 2012)
			Escherichia coli	(Burgess et al. 1973; Karimi Zarchi and Vatani 2009; Roth and Willis 1960)
			Klebsiella aerogenes, K. pneumoniae, K. oxytoca	(Burgess et al. 1973; García et al. 2012; Karimi Zarchi and Vatani 2009; Roth and Willis 1960)
			Morganella morganii	(Karimi Zarchi and Vatani 2009)
			Proteus vulgaris, P. mirabilis	(Burgess et al. 1973; Karimi Zarchi and Vatani 2009; Roth and Willis 1960)
			Salmonella thyphimurium	(Roth and Willis 1960)
			Serratia marcescens	(Burgess et al. 1973; García et al. 2012; Roth and Willis 1960)
			Shimwellia blattae	(Burgess et al. 1973)
		Moraxallaceae	*Acinetobacter calcoaceticus, A. lwoffii*	(Burgess et al. 1973; Ozdal et al. 2016)
		Pseudomonadaceae	*Pseudomonas aeruginosa, P. fluorescens P. putida*	(Burgess et al. 1973; Karimi Zarchi and Vatani 2009; Menasria et al. 2015; Ozdal et al. 2016; Roth and Willis 1960)
		Spirillaceae	*Spirillum* sp.	(Roth and Willis 1960)
		Vibrionaceae	*Vibrio cholerae, V. metschinikovii*	(Roth and Willis 1960)
		Yersiniaceae	*Yersinia pestis*	(Roth and Willis 1960)
		Xanthomonaceae	*Stenotrophomonas maltophilia*	(Ozdal et al. 2016)
	Spirochaetes	*Spirochaetaceae*	*Treponema* spp.	(Roth and Willis 1960)
Blattella germanica (Ectobiidae)	*Actinobacteria*	*Actinomycetaceae*	*Actinomyces radingae*	(Mpuchane et al. 2006b)
		Brevibacteriaceae	*Brevibacterium* spp.	(Mpuchane et al. 2006a; Mpuchane et al. 2006b)
		Corynebacteriaceae	*Corynebacterium* spp.	(Mpuchane et al. 2006a; Mpuchane et al. 2006b)
		Microbacteriaceae	*Aureobacterium* spp.	(Mpuchane et al. 2006b)
		Micrococcaceae	*Arthrobacter* sp.	(Mpuchane et al. 2006a; Mpuchane et al. 2006b)
			Micrococcus aurantiacus, M. luteus	(Fotedar et al. 1991; Gliniewicz et al. 2003; Mpuchane et al. 2006a; Mpuchane et al. 2006b; Roth and Willis 1960)
			Pseudoglutamicibacter cumminsii	(Mpuchane et al. 2006b)
		Mycobacteriaceae	*Mycobacterium leprae*	(Roth and Willis 1960)
		Nocardiaceae	*Rhodococcus australis, R. rhodochrous*	(Mpuchane et al. 2006a; Mpuchane et al. 2006b)
		Tsukamurellaceae	*Tsukamurella inchonensis*	(Mpuchane et al. 2006b)
	Firmicutes	*Bacillaceae*	*Bacillus cereus, B. circulans, B. subtilis, B. thuringiensis*	(Akinjogunla et al. 2012; Fakoorziba et al. 2014; Fotedar et al. 1991; Huang et al. 2013; Karimi Zarchi and Vatani 2009; Mpuchane et al. 2006a; Mpuchane et al. 2006b; Naher et al. 2018; Roth and Willis 1960; Tachbele et al. 2006; Zhang et al. 2018)
		Enterococcaceae	*Enterococcus avium, E. durans, E. faecalis*	(Fakoorziba et al. 2014; Fotedar et al. 1991; Gliniewicz et al. 2003; Jalil et al. 2012; Naher et al. 2018; Roth and Willis 1960; Salehzadeh et al. 2007)
		Leuconostocaceae	*Leuconostoc* sp.	(Mpuchane et al. 2006a; Mpuchane et al. 2006b)
		Paenibacillaceae	*Paenibacillus alvei, P. lentimorbus*	(Strand and Brooks 1977)
		Streptococcaceae	*Streptococcus vestibularis, S. salivarius, S. viridans*	(Elgderi et al. 2006; Fotedar et al. 1991; Gliniewicz et al. 2003; Karimi Zarchi and Vatani 2009; Naher et al. 2018; Salehzadeh et al. 2007; Strand and Brooks 1977; Tilahun et al. 2012)
		Staphylococcaceae	*Staphylococcus aureus, S. equorum, S. epidermidis, S. hominis, S. saprophyticus*	(Abdolmaleki et al. 2019; Akinjogunla et al. 2012; Gliniewicz et al. 2003; Jalil et al. 2012; Karimi Zarchi and Vatani 2009; Menasria et al. 2014; Menasria et al. 2015; Mpuchane et al. 2006a; Mpuchane et al. 2006b; Naher et al. 2018; Oliva et al. 2010; Roth and Willis 1960; Salehzadeh et al. 2007; Tilahun et al. 2012)
	Proteobacteria	*Aeromonadaceae*	*Aeromonas caviae, A. hydrophila*	(Cloarec et al. 1992; Elgderi et al. 2006)
		Alcaligenaceae	*Alcaligenes faecalis*	(Roth and Willis 1960)
		Burkholderiaceae	*Burkholderia cepacia*	(Mpuchane et al. 2006a)
		Comamonadaceae	*Delftia sp.*	(Zhang et al. 2018)

		Enterobacteriaceae	*Buttiauxella agrestis*	(Cloarec et al. 1992; Elgderi et al. 2006; Mpuchane et al. 2006a; Mpuchane et al. 2006b)
			Citrobacter amalonaticus, C. braakii, C. diversus, C. freundii, C. koseri, C. youngae	(Akinjogunla et al. 2012; Cloarec et al. 1992; Elgderi et al. 2006; Fakoorziba et al. 2014; Gliniewicz et al. 2003; Jalil et al. 2012; Karimi Zarchi and Vatani 2009; Menasria et al. 2014; Mpuchane et al. 2006a; Mpuchane et al. 2006b; Oliva et al. 2010; Pai et al. 2005; Roth and Willis 1960; Tilahun et al. 2012; Wannigama et al. 2014)
			Enterobacter agglomerans, E. cloacae, E. gergoviae	(Akinjogunla et al. 2012; Cloarec et al. 1992; Elgderi et al. 2006; Fakoorziba et al. 2014; Fotedar et al. 1991; Gliniewicz et al. 2003; Jalil et al. 2012; Jeffery et al. 2012; Menasria et al. 2014; Mpuchane et al. 2006a; Mpuchane et al. 2006b; Naher et al. 2018; Oliva et al. 2010; Oothuman et al. 1989; Pai et al. 2005; Roth and Willis 1960; Salehzadeh et al. 2007; Tilahun et al. 2012; Vahabi et al. 2011; Wannigama et al. 2014)
			Erwinia spp.	(Mpuchane et al. 2006a; Mpuchane et al. 2006b)
			Escherichia adecarboxylata, E. coli, E. hermannii, E. vulneris	(Akinjogunla et al. 2012; Cloarec et al. 1992; Elgderi et al. 2006; Fakoorziba et al. 2014; Fotedar et al. 1991; Jalil et al. 2012; Karimi Zarchi and Vatani 2009; Mpuchane et al. 2006a; Mpuchane et al. 2006b; Naher et al. 2018; Oliva et al. 2010; Oothuman et al. 1989; Pai et al. 2005; Roth and Willis 1960; Salehzadeh et al. 2007; Strand and Brooks 1977; Tachbele et al. 2006; Tilahun et al. 2012; Vahabi et al. 2011; Wannigama et al. 2014)
			Ewingella americana	(Cloarec et al. 1992)
			Hafnia alvei	(Elgderi et al. 2006; Mpuchane et al. 2006a; Mpuchane et al. 2006b)
			Klebsiella aerogenes, K. oxytoca, K. ozaenae, K. pneumoniae, K. ornithinolytica	(Akinjogunla et al. 2012; Cloarec et al. 1992; Elgderi et al. 2006; Fakoorziba et al. 2014; Fotedar et al. 1991; Gliniewicz et al. 2003; Jalil et al. 2012; Jeffery et al. 2012; Karimi Zarchi and Vatani 2009; Menasria et al. 2014; Mpuchane et al. 2006a; Mpuchane et al. 2006b; Naher et al. 2018; Oliva et al. 2010; Oothuman et al. 1989; Pai et al. 2005; Roth and Willis 1960; Salehzadeh et al. 2007; Strand and Brooks 1977; Tilahun et al. 2012; Vahabi et al. 2011; Wannigama et al. 2014)
			Kluyvera intermedia	(Cloarec et al. 1992; Mpuchane et al. 2006a; Mpuchane et al. 2006b)
			Leclercia adecarboxylata	(Elgderi et al. 2006)
			Morganella sp., *Morganella morganii*	(Akinjogunla et al. 2012; Cloarec et al. 1992; Elgderi et al. 2006; Jalil et al. 2012; Oothuman et al. 1989)
			Pantoea agglomerans	(Elgderi et al. 2006; Menasria et al. 2014)
			Proteus mirabilis, P. vulgaris	(Akinjogunla et al. 2012; Elgderi et al. 2006; Fakoorziba et al. 2014; Fotedar et al. 1991; Jalil et al. 2012; Karimi Zarchi and Vatani 2009; Mpuchane et al. 2006a; Mpuchane et al. 2006b; Naher et al. 2018; Pai et al. 2005; Vahabi et al. 2011; Wannigama et al. 2014)
			Providencia alcalifaciens, P. rettgeri	(Akinjogunla et al. 2012; Cloarec et al. 1992; Jalil et al. 2012; Tilahun et al. 2012)
			Salmonella enterica, S. typhimurium	(Akinjogunla et al. 2012; Fathpour et al. 2003; García et al. 2012; Jalil et al. 2012; Mpuchane et al. 2006a; Mpuchane et al. 2006b; Naher et al. 2018; Oothuman et al. 1989; Roth and Willis 1960; Tachbele et al. 2006; Tilahun et al. 2012; Wannigama et al. 2014)
			Serratia liquefaciens, S. marcescens, S. odorifera, S. plymuthica, S. rubidaea	(Cloarec et al. 1992; Elgderi et al. 2006; Fotedar et al. 1991; Gliniewicz et al. 2003; Jalil et al. 2012; Jeffery et al. 2012; Menasria et al. 2014; Mpuchane et al. 2006a; Mpuchane et al. 2006b; Oliva et al. 2010; Oothuman et al. 1989; Pai et al. 2005; Roth and Willis 1960; Strand and Brooks 1977; Vahabi et al. 2011)
			Shigella flexneri	(Akinjogunla et al. 2012; Fakoorziba et al. 2014; Jalil et al. 2012; Mpuchane et al. 2006b; Naher et al. 2018; Salehzadeh et al. 2007; Tachbele et al. 2006; Tilahun et al. 2012)
		Moraxellaceae	*Acinetobacter spp.*	(Cloarec et al. 1992; Elgderi et al. 2006; Fakoorziba et al. 2014; Oliva et al. 2010; Tilahun et al. 2012; Zhang et al. 2018)
		Pasteurellaceae	*Haemophilus* sp.	(Naher et al. 2018; Salehzadeh et al. 2007)
		Pseudomonadaceae	*Pseudomonas aeruginosa, P. fluorescens, P. putida, P. reactans*	(Akinjogunla et al. 2012; Cloarec et al. 1992; Elgderi et al. 2006; Fakoorziba et al. 2014; Fotedar et al. 1991; Gliniewicz et al. 2003; Jalil et al. 2012; Karimi Zarchi and Vatani 2009; Loucif et al. 2017; Menasria et al. 2014; Menasria et al. 2015; Mpuchane et al. 2006a; Mpuchane et al. 2006b;

				Naher et al. 2018; Oothuman et al. 1989; Roth and Willis 1960; Saitou et al. 2009; Salehzadeh et al. 2007; Strand and Brooks 1977; Tilahun et al. 2012; Vahabi et al. 2011; Wannigama et al. 2014; Zhang et al. 2013; Zhang et al. 2018)
		Vibrionaceae	*Vibrio cholerae, V. fluvialis, V. metschinikovii*	(Cloarec et al. 1992; Mpuchane et al. 2006b; Roth and Willis 1960)
		Xanthomonaceae	*Stenotrophomonas maltophilia*	(Elgderi et al. 2006; Mpuchane et al. 2006b)
			Xanthomonas spp.	(Mpuchane et al. 2006a; Mpuchane et al. 2006b)
Cryptocercus punctulatus (Cryptocercidae)	*Firmicutes*	*Bacillaceae*	*Bacillus subtilis*	(Roth and Willis 1960)
	Spirochaetes	*Spirochaetaceae*	*Alkalispirochaeta cellulosivorans*	(Sravanthi et al. 2016)
Diploptera punctata (Blaberidae)	*Proteobacteria*	*Enterobacteriaceae*	*Serratia marcescens*	(Roth and Willis 1960)
Eublaberus posticus (Blaberidae)	*Actinobacteria*	*Coriobacteriaceae*	*Collinsella aerofaciens*	(Cruden and Markovetz 1987)
		Propionibacteriaceae	*Cutibacterium avidum*	(Cruden and Markovetz 1987)
			Propionibacterium freudenreichii	(Cruden and Markovetz 1987)
	Firmicutes	*Clostridiaceae*	*Clostridium beijerinckii, C. carnis, C. moniliforme, C. sporogenes*	(Cruden and Markovetz 1987; Cruden and Markovetz 1979)
			Paenoclostridium sp.	(Cruden and Markovetz 1987)
			Paraclostridium bifermentans	(Cruden and Markovetz 1987)
		Eubacteriaceae	*Eubacterium cellulosolvens, E. limosum*	(Cruden and Markovetz 1987; Cruden and Markovetz 1979)
		Lachnospiraceae	*Blautia producta*	(Cruden and Markovetz 1987)
		Oscillospiraceae	*Faecalibacterium prausnitzii*	(Foglesong et al. 1984)
		Peptoniphilaceae	*Finegoldia magna*	(Cruden and Markovetz 1987)
		Peptostreptococcaceae	*Clostridioides mangenotii*	(Cruden and Markovetz 1987)
			Peptostreptococcus anaerobius	(Cruden and Markovetz 1987)
		Streptococcaceae	*Streptococcus constellatus, S. intermedius*	(Cruden and Markovetz 1987)
		Tissierallaceae	*Tissierella preacuta*	(Cruden and Markovetz 1987)
	Fusobacteria	*Fusobacteriaceae*	*Fusobacterium gonidiaformans, F. necrophorum, F. prausnitzii, F. varium*	(Cruden and Markovetz 1987; Foglesong et al. 1984)
	Proteobacteria	*Enterobacteriaceae*	*Citrobacter freundii*	(Cruden and Markovetz 1979)
			Klebsiella pneumoniae	(Cruden and Markovetz 1979)
			Serratia sp.	(Cruden and Markovetz 1979)
Gromphadorhina portentosa (Blaberidae)	*Firmicutes*	*Bacillaceae*	*Bacillus cereus*	(Margulis et al. 1998)
		Enterococcaceae	*Enterococcus* sp.	(Robertson 2007)
	Fusobacteria	*Fusobacteriaceae*	*Fusobacterium* sp.	(Robertson 2007)
	Proteobacteria	*Enterobacteriaceae*	*Klebsiella* sp.	(Robertson 2007)
			Serratia sp.	(Robertson 2007)
		Pseudomonadaceae	*Pseudomonas* sp.	(Robertson 2007)
Panchlora nivea (Blaberidae)	*Proteobacteria*	*Enterobacteriaceae*	*Serratia marcescens*	(Roth and Willis 1960)
Periplaneta americana (Blattidae)	*Actinobacteria*	*Bifidobacteriaceae*	*Bifidobacterium* sp.	(Cruden and Markovetz 1987)
		Corynebacteriaceae	*Corynebacterium* sp.	(Strand and Brooks 1977)
		Micrococcaceae	*Micrococcus* spp.	(Kassiri et al. 2014)
		Mycobacteriaceae	*Mycobacterium aurum, M. avium, M. fortuitum, M. gordonae, M. kansasii, M.*	(Leibovitz 1951; Pai et al. 2003; Roth and Willis 1960)

			lacticola, M. leprae, M. phlei, M. piscium, M. ranae, M. smegmatis, M. xenopii	
		Nocardiaceae	*Nocardia* sp.	(Leibovitz 1951)
		Propionibacteriaceae	*Cutibacterium avidum*	(Cruden and Markovetz 1987)
		Streptomycetaceae	*Streptomyces globisporus*	(Chen et al. 2020; Hoffman 1953)
	Bacteroidetes	*Bacteroidaceae*	*Bacteroides* spp.	(Cruden and Markovetz 1987; Vera-Ponce de León et al. 2020)
		Dysgonomonadaceae	*Dysgonomonas* spp.	(Vera-Ponce de León et al. 2020)
		Flavobacteriaceae	*Chryseobacterium* sp.	(Dugas et al. 2001)
		Paludibacteraceae	*Paludibacter* sp.	(Vera-Ponce de León et al. 2020)
		Tannerellaceae	*Parabacteroides* sp.	(Vera-Ponce de León et al. 2020)
	Firmicutes	*Acidaminococcaceae*	*Acidaminococcus fermentans*	(Cruden and Markovetz 1987)
		Bacillaceae	*Bacillus cereus, B. kochii, B. megaterium, B. subtilis, B. thuringiensis*	(Akinjogunla et al. 2012; Al-Fattly and Al-Aridhi 2014; Alcamo and Frishman 1980; Bagde et al. 2013; Elyasigomari et al. 2017; Feizhaddad et al. 2012; Isaac et al. 2014; Karimi Zarchi and Vatani 2009; Oothuman et al. 1989; Rampal et al. 1983; Roth and Willis 1960; Sayyad et al. 2016; Sharma et al. 2019; Strand and Brooks 1977)
		Clostridiaceae	*Clostridium moniliforme, C. sporogenes*	(Cruden and Markovetz 1987)
			Paraclostridium bifermentans	(Cruden and Markovetz 1987)
			Sarcina sp.	(Roth and Willis 1960)
		Enterococcaceae	*Enterococcus faecalis*	(Al-Fattly and Al-Aridhi 2014; Bouamamaa et al. 2010; Isaac et al. 2014; Jalil et al. 2012; Kane and Breznak 1991; Kassiri et al. 2014; Roth and Willis 1960; Tetteh-Quarcoo et al. 2013)
		Lachnospiraceae	*Blautia producta*	(Cruden and Markovetz 1987)
			Butyrivibrio sp.	(Cruden and Markovetz 1987)
			Coprococcus sp.	(Cruden and Markovetz 1987)
		Lactobacillaceae	*Lactobacillus* sp.	(Cruden and Markovetz 1987; Kane and Breznak 1991)
		Peptoniphilaceae	*Finegoldia magna*	(Cruden and Markovetz 1987)
		Oscillospiraceae	*Ruminococcus* sp.	(Cruden and Markovetz 1987)
		Streptococcaceae	*Streptococcus pyogenes*	(Al-Fattly and Al-Aridhi 2014; Alcamo and Frishman 1980; Feizhaddad et al. 2012; Kane and Breznak 1991; Karimi Zarchi and Vatani 2009; Kassiri et al. 2014; Oothuman et al. 1989; Rampal et al. 1983)
		Staphylococcaceae	*Staphylococcus aureus, S. epidermidis, S. saprophyticus*	(Abdolmaleki et al. 2019; Akinjogunla et al. 2012; Al-Fattly and Al-Aridhi 2014; Alcamo and Frishman 1980; Bagde et al. 2013; Bouamamaa et al. 2010; Elyasigomari et al. 2017; Feizhaddad et al. 2012; Isaac et al. 2014; Islam et al. 2016; Jalil et al. 2012; Karimi Zarchi and Vatani 2009; Kassiri et al. 2014; Oothuman et al. 1989; Prado et al. 2006; Rampal et al. 1983; Sayyad et al. 2016; Vahabi et al. 2011)
		Veillonellaceae	*Veillonella parvula*	(Roth and Willis 1960)
	Fusobacteria	*Fusobacteriaceae*	*Fusobacterium varium*	(Cruden and Markovetz 1987)
	Proteobacteria	*Alcaligenaceae*	*Alcaligenes faecalis*	(Roth and Willis 1960)
		Burkholderiaceae	*Burkholderia cepacia*	(García et al. 2012)
		Enterobacteriaceae	*Citrobacter diversus, C. freundii, C. koseri, C. intermedius*	(Akbari et al. 2015; Bouamamaa et al. 2010; Elyasigomari et al. 2017; García et al. 2012; Iboh et al. 2014; Isaac et al. 2014; Jalil et al. 2012; Jeffery et al. 2012; Karimi Zarchi and Vatani 2009; Kassiri et al. 2014; Oothuman et al. 1989; Pai et al. 2005; Rampal et al. 1983; Roth and Willis 1960; Tetteh-Quarcoo et al. 2013; Wannigama et al. 2014)
			Enterobacter cloacae, E. aerogenes	(Akbari et al. 2015; Akinjogunla et al. 2012; Bagde et al. 2013; Bouamamaa et al. 2010; Elyasigomari et al. 2017; Fakoorziba et al. 2014; Feizhaddad et al. 2012; García et al. 2012; Iboh et al. 2014; Jalil et al. 2012; Jeffery et al. 2012; Karimi Zarchi and Vatani 2009; Kassiri et al. 2014; Oothuman et al. 1989; Pai et al. 2005; Prado et al. 2006; Rampal et al. 1983; Roth and Willis 1960; Sayyad et al. 2016; Tetteh-Quarcoo et al. 2013; Vahabi et al. 2011; Wannigama et al. 2014)

			Escherichia coli, E. vulneris	(Akbari et al. 2015; Akinjogunla et al. 2012; Al-Fattly and Al-Aridhi 2014; Bagde et al. 2013; Bouamamaa et al. 2010; Elyasigomari et al. 2017; Feizhaddad et al. 2012; García et al. 2012; Isaac et al. 2014; Jalil et al. 2012; Jeffery et al. 2012; Karimi Zarchi and Vatani 2009; Kassiri et al. 2014; Oothuman et al. 1989; Pai et al. 2005; Rampal et al. 1983; Roth and Willis 1960; Sayyad et al. 2016; Strand and Brooks 1977; Tetteh-Quarcoo et al. 2013; Vahabi et al. 2011; Wannigama et al. 2014)
			Edwardsiella tarda	(Karimi Zarchi and Vatani 2009)
			Hafnia alvei	(García et al. 2012; Pai et al. 2005; Prado et al. 2006)
			Klebsiella aerogenes, K. pneumoniae, K. oxytoca	(Akbari et al. 2015; Akinjogunla et al. 2012; Al-Fattly and Al-Aridhi 2014; Bouamamaa et al. 2010; García et al. 2012; Iboh et al. 2014; Jalil et al. 2012; Jeffery et al. 2012; Karimi Zarchi and Vatani 2009; Kassiri et al. 2014; Oothuman et al. 1989; Pai et al. 2005; Prado et al. 2006; Rampal et al. 1983; Roth and Willis 1960; Sayyad et al. 2016; Tetteh-Quarcoo et al. 2013; Vahabi et al. 2011; Wannigama et al. 2014)
			Leclercia adecarboxylata	(Akbari et al. 2015)
			Morganella morganii	(Akinjogunla et al. 2012; García et al. 2012; Jalil et al. 2012; Oothuman et al. 1989; Roth and Willis 1960)
			Proteus mirabilis, P. vulgaris	(Agbodaze 1989; Akinjogunla et al. 2012; Al-Fattly and Al-Aridhi 2014; Bouamamaa et al. 2010; Elyasigomari et al. 2017; Feizhaddad et al. 2012; García et al. 2012; Isaac et al. 2014; Jalil et al. 2012; Jeffery et al. 2012; Karimi Zarchi and Vatani 2009; Kassiri et al. 2014; Oothuman et al. 1989; Pai et al. 2005; Rampal et al. 1983; Roth and Willis 1960; Sayyad et al. 2016; Tetteh-Quarcoo et al. 2013; Vahabi et al. 2011; Wannigama et al. 2014)
			Providencia rettgeri	(Akinjogunla et al. 2012; Bouamamaa et al. 2010; Elyasigomari et al. 2017; Jalil et al. 2012; Oothuman et al. 1989; Roth and Willis 1960)
			Rahnella aquatilis	(Akbari et al. 2015)
			Salmonella enterica, S. typhimurium	(Agbodaze 1989; Akinjogunla et al. 2012; Al-Fattly and Al-Aridhi 2014; Bagde et al. 2013; Bouamamaa et al. 2010; Elyasigomari et al. 2017; Iboh et al. 2014; Isaac et al. 2014; Jalil et al. 2012; Rampal et al. 1983; Roth and Willis 1960; Wannigama et al. 2014)
			Serratia marcescens, S. odorifera, S. rubidaea	(Bouamamaa et al. 2010; Elyasigomari et al. 2017; Feizhaddad et al. 2012; Kassiri et al. 2014; Pai et al. 2005; Prado et al. 2006; Sayyad et al. 2016; Strand and Brooks 1977; Vahabi et al. 2011)
			Shigella boydii, S. dysenteriae, S. flexneri, S. sonnei	(Agbodaze 1989; Akbari et al. 2015; Akinjogunla et al. 2012; Al-Fattly and Al-Aridhi 2014; Bagde et al. 2013; Bouamamaa et al. 2010; Elyasigomari et al. 2017; Iboh et al. 2014; Jalil et al. 2012; Oothuman et al. 1989; Rampal et al. 1983)
		Moraxellaceae	*Acinetobacter* sp.	(Bouamamaa et al. 2010; Oothuman et al. 1989; Rampal et al. 1983)
		Pasteurellaceae	*Pasteurella* sp.	(Bouamamaa et al. 2010)
		Pseudomonadaceae	*Pseudomonas aeruginosa, P. fluorescens*	(Agbodaze 1989; Akinjogunla et al. 2012; Al-Fattly and Al-Aridhi 2014; Fakoorziba et al. 2014; Isaac et al. 2014; Jalil et al. 2012; Karimi Zarchi and Vatani 2009; Kassiri et al. 2014; Oothuman et al. 1989; Rampal et al. 1983; Roth and Willis 1960; Saitou et al. 2009; Sayyad et al. 2016; Strand and Brooks 1977; Tetteh-Quarcoo et al. 2013; Vahabi et al. 2011; Wannigama et al. 2014)
		Spirillaceae	*Spirillum* sp.	(Roth and Willis 1960)
		Vibrionaceae	*Vibrio cholerae*	(Iboh et al. 2014; Roth and Willis 1960)
		Yersiniaceae	*Yersinia enterocolitica, Y. intermedia, Y. pseudotuberculosis*	(Akbari et al. 2015; Bouamamaa et al. 2010)
	Spirochaetes	*Leptospiraceae*	*Leptospira* sp.	(Gonzalez-Astudillo et al. 2015)
Periplaneta australasiae (Blattidae)	*Actinobacteria*	*Mycobacteriaceae*	*Mycobacterium leprae*	(Roth and Willis 1960)
	Firmicutes	*Bacillaceae*	*Bacillus* sp.	(Rampal et al. 1983)
	Proteobacteria	*Enterobacteriaceae*	*Enterobacter* sp.	(Rampal et al. 1983)

			Serratia marcescens	(Roth and Willis 1960)
		Vibrionaceae	*Vibrio cholerae*	(Roth and Willis 1960)
	Spirochaetes	*Leptospiraceae*	*Leptospira* sp.	(Gonzalez-Astudillo et al. 2015)
Periplaneta brunnea (Blattidae)	*Firmicutes*	*Bacillaceae*	*Bacillus* sp.	(Oothuman et al. 1989; Rampal et al. 1983)
		Streptococcaceae	*Streptococcus* spp.	(Oothuman et al. 1989)
	Proteobacteria	*Enterobacteriaceae*	*Citrobacter koseri*	(Jeffery et al. 2012; Rampal et al. 1983)
			Enterobacter sp.	(Rampal et al. 1983)
			Escherichia coli, E. vulneris	(Jeffery et al. 2012; Oothuman et al. 1989; Rampal et al. 1983)
			Klebsiella pneumoniae	(Jeffery et al. 2012; Rampal et al. 1983)
			Salmonella typhimurium	(Oothuman et al. 1989)
			Serratia marcescens	(Roth and Willis 1960)
		Moraxellaceae	*Acinetobacter calcoaceticus*	(Jeffery et al. 2012)
		Pseudomonadaceae	*Pseudomonas* sp.	(Oothuman et al. 1989)
Periplaneta fuliginosa (Blattidae)	*Proteobacteria*	*Enterobacteriaceae*	*Serratia marcescens*	(Rueger and Olson 1969)
		Pseudomonadaceae	*Pseudomonas aeruginosa*	(Saitou et al. 2009)
Polyphaga aegyptiaca (Corydiidae)	*Firmicutes*	*Bacillaceae*	*Bacillus cereus, B. subtilis*	(Elyasigomari et al. 2017; Vazirianzadeh et al. 2009)
		Staphylococcaceae	*Staphylococcus aureus, S. epidermidis*	(Elyasigomari et al. 2017; Vazirianzadeh et al. 2009)
	Proteobacteria	*Alcaligenaceae*	*Oligella urethralis*	(Elyasigomari et al. 2017)
		Burkholderiaceae	*Burkholderia mallei*	(Elyasigomari et al. 2017)
		Enterobacteriaceae	*Citrobacter freundii*	(Elyasigomari et al. 2017)
			Enterobacter cloacae	(Elyasigomari et al. 2017)
			Escherichia coli	(Elyasigomari et al. 2017; Vazirianzadeh et al. 2009)
			Klebsiella pneumoniae	(Vazirianzadeh et al. 2009)
			Morganella morganii	(Elyasigomari et al. 2017)
			Providencia spp.	(Elyasigomari et al. 2017)
			Serratia marcescens	(Elyasigomari et al. 2017)
		Neisseriaceae	*Neisseria* sp.	(Vazirianzadeh et al. 2009)
Pycnoscelus surinamensis (Blaberidae)	*Proteobacteria*	*Enterobacteriaceae*	*Serratia marcescens*	(Roth and Willis 1960)
Nauphoeta cinerea (Blaberidae)	*Firmicutes*	*Bacillaceae*	*Bacillus* sp.	(Rampal et al. 1983)
	Proteobacteria	*Enterobacteriaceae*	*Enterobacter* sp.	(Rampal et al. 1983)
			Salmonella typhimurium	(Roth and Willis 1960)
			Serratia marcescens	(Roth and Willis 1960)
		Moraxellaceae	*Acinetobacter* sp.	(Rampal et al. 1983)
Neostylopyga rhombifolia (Blattidae)	*Firmicutes*	*Bacillaceae*	*Bacillus* sp.	(Rampal et al. 1983)
	Proteobacteria	*Enterobacteriaceae*	*Escherichia coli*	(Rampal et al. 1983)
			Klebsiella sp.	(Rampal et al. 1983)
			Serratia marcescens	(Roth and Willis 1960)
Rhyparobia maderae (Blaberidae)	*Firmicutes*	*Enterococcaceae*	*Enterococcus faecalis*	(Strand and Brooks 1977)
	Proteobacteria	*Enterobacteriaceae*	*Citrobacter* sp.	(Strand and Brooks 1977)
			Hafnia sp.	(Strand and Brooks 1977)
			Serratia marcescens	(Roth and Willis 1960)
	Firmicutes	*Bacillaceae*	*Bacillus thuringiensis*	(Strand and Brooks 1977)
		Erysipelotrichaceae	*Breznakia blatticola*	(Tegtmeier et al. 2016a)

Shelfordella lateralis (Blattidae)	*Fusobacteria*	*Fusobacteriaceae*	*Fusobacterium varium*	(Tegtmeier et al. 2016b)
	Proteobacteria	*Enterobacteriaceae*	*Cronobacter* sp.	(Tegtmeier et al. 2016b)
			Pantoea sp.	(Tegtmeier et al. 2016b)
			Shimwellia sp.	(Tegtmeier et al. 2016b)
	Verrucomicrobia	*Opitutaceae*	*Ereboglobus luteus*	(Tegtmeier et al. 2018)
Supella longipalpa (Ectobiidae)	*Firmicutes*	*Bacillaceae*	*Bacillus cereus, B. subtilis*	(Oothuman et al. 1989; Vazirianzadeh et al. 2014)
		Staphylococcaceae	*Staphylococcus* sp., *S. aureus*	(Alcamo and Frishman 1980; Le Guyader et al. 1989; Oothuman et al. 1989; Strand and Brooks 1977; Vazirianzadeh et al. 2014)
		Streptococcaceae	*Streptococcus* sp.	(Alcamo and Frishman 1980)
	Proteobacteria	*Aeromonadaceae*	*Aeromonas hydrophila*	(Le Guyader et al. 1989)
		Alcaligenaceae	*Achromobacter* spp.	(Le Guyader et al. 1989)
			Alcaligenes faecalis	(Le Guyader et al. 1989)
		Burkholderiaceae	*Burkholderia cepacia*	(Le Guyader et al. 1989)
		Enterobacteriaceae	*Buttiauxella agrestis*	(Le Guyader et al. 1989)
			Cedecea sp.	(Le Guyader et al. 1989)
			Citrobacter koseri, C. freundii	(Le Guyader et al. 1989; Vazirianzadeh et al. 2014)
			Cronobacter sakazakii	(Le Guyader et al. 1989)
			Enterobacter cloacae	(Le Guyader et al. 1989; Vazirianzadeh et al. 2014)
			Escherichia coli	(Le Guyader et al. 1989; Vazirianzadeh et al. 2014)
			Klebsiella aerogenes, K. pneumoniae, K. oxytoca	(Le Guyader et al. 1989; Vazirianzadeh et al. 2014)
			Kluyvera sp.	(Le Guyader et al. 1989)
			Leclercia adecarboxylata	(Le Guyader et al. 1989)
			Lelliottia amnigena	(Le Guyader et al. 1989)
			Pantoea agglomerans	(Le Guyader et al. 1989)
			Proteus mirabilis, P. vulgaris	(Le Guyader et al. 1989; Vazirianzadeh et al. 2014)
			Serratia liquefaciens, S. marcescens	(Le Guyader et al. 1989; Roth and Willis 1960; Vazirianzadeh et al. 2014)
			Shigella boydii, S. dysenteriae, S. flexneri, S. sonnei	(Le Guyader et al. 1989)
		Moraxellaceae	*Acinetobacter calcoaceticus*	(Le Guyader et al. 1989)
		Pseudomonadaceae	*Pseudomonas aeruginosa, P. fluorescens, P. stutzeri*	(Le Guyader et al. 1989)
		Sphingomonadaceae	*Sphingomonas paucimobilis*	(Le Guyader et al. 1989)
		Xanthomonadaceae	*Stenotrophomonas maltophilia*	(Le Guyader et al. 1989)

Online Resource 3. Average composition of the bacterial community of wild or untreated cockroaches at the level of bacterial phyla.

Cockroach		16S rRNA region	Sequencing platform	Taxonomic database	Composition by phylum (%)											References
					Actinob.	Bactero.	Firmicu.	Fusobac.	Plancto.	Proteob.	Spiroch.	Synergi.	Teneric.	Verruc.	Other	
Blaberus craniifer		515F-806R	Illumina MiSeq	Greengenes 2013	1.3	26	34	2.8	0.9	18	0.2	1.4	2.4	6.1	6.9	(Tinker and Ottesen 2020)
Blatta orientalis		343F-784R	454 Roche pyroseq.	DictDb v.2.3	0.5	43	38	0.7	1.4	8.8	1.1	0.9	0.0	1.2	4.4	(Dietrich et al. 2014)
Blattella germanica	A	8F-530R	454 Roche pyroseq.	No data	0.2	41	24	4.0	0.0	30	0.0	0.2	0.0	0.4	0.2	(Pérez-Cobas et al. 2015)
	B	515F-806R	Illumina MiSeq	Greengenes 13.8	0.2	48	25	0.4	1.6	20	0.03	0.2	0.7	0.1	3.8	(Kakumanu et al. 2018)
	C	V4 region	Illumina MiSeq	Greengenes 13.8	0.00	33	10	0.0	0.0	47	0.0	0.0	0.0	2.1	7.9	(Pietri et al. 2018)
	D	V3-V4 region	Illumina MiSeq	SILVA v.119	0.2	48	19	4.2	1.9	17	0.0	0.0	0.0	0.0	9.8	(Rosas et al. 2018)
	E	515F-806R	Illumina MiSeq	Greengenes 2013	1.3	28	28	6.5	3.8	23	0.3	1.7	0.8	0.7	5.9	(Tinker and Ottesen 2020)
Byrsotria fumigata		343F-784R	Illumina MiSeq	DictDb v.3.0	1.7	23	41	0.0	0.8	6.6	1.7	0.8	1.7	0.0	23	(Mikaelyan et al. 2015)
Cryptocercus punctulatus	A	343F-784R	454 Roche pyroseq.	DictDb v.2.3	4.8	30	40	0.0	2.7	4.4	8.4	0.8	0.0	1.2	7.7	(Dietrich et al. 2014)
Cryptocercus punctulatus	B	8F-1391R	Sanger	GenBank	7.4	26	33	0.0	0.0	19	3.2	2.6	1.8	2.1	4.9	(Berlanga et al. 2009)
Diploptera punctata	A	343F-784R	454 Roche pyroseq.	DictDb v.2.3	0.5	31	36	7.1	2.9	14	0.04	6.0	0.0	0.4	2.1	(Dietrich et al. 2014)
Diploptera punctata	B	515F-806R	Illumina MiSeq	SILVA v.123	1.9	27	38	0.9	1.7	15	0.04	1.5	1.2	0.3	12	(Jennings et al. 2019)
Diploptera punctata	C	515F-806R	Illumina MiSeq	Greengenes 2013	1.5	26	35	0.8	1.3	23	0.2	7.3	0.5	0.3	4.1	(Tinker and Ottesen 2020)
Elliptorhina chopardi		343F-784R	454 Roche pyroseq.	DictDb v.2.3	0.5	35	47	2.3	1.3	9.4	0.7	1.0	0.0	0.0	2.8	(Dietrich et al. 2014)
Ergaula capucina	A	343F-784R	454 Roche pyroseq.	DictDb v.2.3	2.0	23	56	0.3	1.6	12	1.0	0.8	0.0	0.0	3.2	(Dietrich et al. 2014)
Ergaula capucina	B	343F-784R	Illumina MiSeq	DictDb v.3.0	0.0	28	40	0.0	0.0	16	8.0	0.0	0.0	0.0	8.0	(Mikaelyan et al. 2015)
Ergaula capucina	C	515F-806R	Illumina MiSeq	Greengenes 2013	5.1	17	51	1.0	1.9	14	0.3	0.8	1.2	0.1	6.6	(Tinker and Ottesen 2020)
Ergaula pilosa		515F-806R	Illumina MiSeq	Greengenes 2013	3.5	12	49	0.2	4.0	13	0.3	2.2	2.6	0.6	6.5	(Tinker and Ottesen 2020)

Eublaberus posticus		343F-784R	454 Roche pyroseq.	DictDb v.2.3	3.3	24	45	2.1	2.8	8.8	1.8	1.8	0.0	0.2	10.2	(Dietrich et al. 2014)
Eurycotis floridana		343F-784R	454 Roche pyroseq.	DictDb v.2.3	0.2	39	42	2.6	2.0	8.2	0.8	0.4	0.0	1.3	3.5	(Dietrich et al. 2014)
Gromphadorhina portentosa		515F-806R	Illumina MiSeq	Greengenes 2013	1.1	30	31	1.0	1.5	24	0.2	3.1	1.0	0.6	10.1	(Tinker and Ottesen 2020)
Lucihormetica verrucosa		515F-806R	Illumina MiSeq	Greengenes 2013	5.8	17	36	3.1	1.1	23	0.7	0.8	1.8	1.4	5.9	(Tinker and Ottesen 2020)
Nauphoeta cinerea		515F-806R	Illumina MiSeq	Greengenes 2013	3.6	39	24	0.6	4.2	17	0.6	3.1	0.6	1.0	6.4	(Tinker and Ottesen 2020)
Opisthoplatia orientalis		343F-784R	454 Roche pyroseq.	DictDb v.2.3	1.4	27	32	3.1	3.8	14	1.9	8.8	0.0	2.5	5.5	(Dietrich et al. 2014)
Oxyhaloa deusta		515F-806R	Illumina MiSeq	Greengenes 2013	1.8	29	26	2.1	2.8	25	0.1	4.7	1.1	2.2	8.5	(Tinker and Ottesen 2020)
Panchlora sp.		343F-784R	454 Roche pyroseq.	DictDb v.2.3	0.7	36	18	3.7	0.9	27	0.6	0.4	0.0	0.1	13	(Dietrich et al. 2014)
Panchlora viridis		515F-806R	Illumina MiSeq	Greengenes 2013	3.7	19	20	1.0	3.0	38	1.3	0.3	3.0	0.0	3.9	(Tinker and Ottesen 2020)
Panesthia angustipennis	A	343F-784R	454 Roche pyroseq.	DictDb v.2.3	0.4	33	41	0.2	2.6	3.5	0.9	7.0	0.0	3.0	8.4	(Dietrich et al. 2014)
Panesthia angustipennis	B	343F-784R	Illumina MiSeq	DictDb v.3.0	3.0	40	36	0.0	3.8	11	0.0	0.8	1.5	0.0	3.9	(Mikaelyan et al. 2015)
Paraplecta sp. "Kenya"		515F-806R	Illumina MiSeq	Greengenes 2013	2.9	21	30	1.8	2.7	30	0.2	1.1	1.2	0.6	2.9	(Tinker and Ottesen 2020)
Parcoblatta fulvescens		515F-806R	Illumina MiSeq	Greengenes 2013	0.9	35	34	4.4	1.6	19	0.04	0.6	1.0	0.0	5.3	(Tinker and Ottesen 2020)
Periplaneta americana	A	27F-907R	Sanger	No data	0.0	27	47	0.0	0.0	19	0.0	1.7	0.0	0.3	10	(Bertino-Grimaldi et al. 2013)
	B	515F-806R	Illumina MiSeq	Greengenes 2013	0.0	43	32	0.5	0.4	11	0.0	0.8	1.8	0.5	8.7	(Tinker and Ottesen 2016)
	C	515F-806R	Illumina MiSeq	Greengenes 2013	0.9	32	35	0.3	1.8	18	0.3	1.3	1.2	0.5	6.9	(Tinker and Ottesen 2020)
Periplaneta fuliginosa		515F-806R	Illumina MiSeq	Greengenes 2013	0.1	42	24	2.8	2.6	18	0.2	1.7	1.2	0.5	3.4	(Tinker and Ottesen 2020)
Periplaneta japonica		V3-V4 region	Illumina MiSeq	No data	0.6	37	40	2.0	0.5	15	0.1	0.08	0.8	0.4	6.9	(Leite-Vicente et al. 2018)
Polyphaga aegyptiaca		515F-806R	Illumina MiSeq	Greengenes 2013	1.5	23	44	0.4	2.0	17	0.2	1.8	0.6	0.6	8.9	(Tinker and Ottesen 2020)
Pycnoscelus surinamensis	A	343F-784R	Illumina MiSeq	DictDb v.3.0	4.0	23	36	0.0	1.0	17	0.0	1.0	1.0	0.0	17	(Mikaelyan et al. 2015)
	B	504F-711R	Illumina MiSeq	DictDb v.3.0	4.1	27	39	0.3	6.5	15	0.1	5.1	0.1	0.7	2.1	(Richards et al. 2017)

	C	515F-806R	Illumina MiSeq	Greengenes 2013	5.2	20	34	4.2	1.1	26	0.3	2.0	2.0	0.5	4.7	(Tinker and Ottesen 2020)
Rhyparobia maderae		343F-784R	454 Roche pyroseq.	DictDb v.2.3	0.6	31	36	5.5	1.7	14	1.1	4.7	0.0	2.3	3.1	(Dietrich et al. 2014)
Salganea esakii		343F-784R	454 Roche pyroseq.	DictDb v.2.3	0.6	36	42	0.2	1.6	4.3	1.1	2.7	0.0	1.2	10.3	(Dietrich et al. 2014)
Schultesia lampyridiformis	A	343F-784R	454 Roche pyroseq.	DictDb v.2.3	0.6	43	32	2.2	2.4	13	1.1	2.2	0.0	0.4	3.1	(Dietrich et al. 2014)
Schultesia lampyridiformis	B	515F-806R	Illumina MiSeq	Greengenes 2013	2.2	28	29	2.1	4.1	24	0.1	2.5	0.3	0.7	7.0	(Tinker and Ottesen 2020)
Shelfordella lateralis	A	343F-784R	454 Roche pyroseq.	DictDb v.2.3	0.5	43	38	0.7	1.4	8.8	1.1	0.9	0.0	1.2	4.5	(Dietrich et al. 2014)
	B	343F-784R	454 Roche pyroseq.	SILVA v.102	2.0	26	44	4.2	2.8	12	0.4	1.6	0.0	2.1	4.9	(Schauer et al. 2014)
	C	343F-784R	Illumina MiSeq	DictDb v.3.0	1.5	37	43	0.8	2.3	9.2	0.0	0.8	1.5	0.0	3.9	(Mikaelyan et al. 2015)
	D	343F-784R	454 Roche pyroseq.	DictDb v.3.0	1.1	42	32	1.5	3.4	12	0.8	0.03	0.2	0.4	6.6	(Mikaelyan et al. 2016)
Symploce macroptera		343F-784R	454 Roche pyroseq.	DictDb v.2.3	0.3	43	29	0.34	5.7	14	1.9	2.6	0.0	0.3	2.4	(Dietrich et al. 2014)
Symploce pallens		515F-806R	Illumina MiSeq	Greengenes 2013	1.4	39	30	3.9	1.1	17	0.2	0.9	2.5	0.8	3.2	(Tinker and Ottesen 2020)
Therea olegrandjeani		515F-806R	Illumina MiSeq	Greengenes 2013	1.5	18	56	3.3	1.3	12	0.1	1.4	0.8	0.3	5.3	(Tinker and Ottesen 2020)

The data presented here was either extracted directly from the supplementary information of the published papers, or was obtained from the authors, and in some cases the data was calculated from the tables and plots in the original publications.

Online Resource 4. Average composition of the bacterial community of cockroaches at the level of bacterial phyla according to the gut compartment (foregut/crop, midgut, hindgut)-

**Cockroach**		**16S rRNA region**	**Sequencing platform**	**Taxonomic database**	**Organ**	**Composition by phyla**											**References**
						**Acti nob.**	**Bact ero.**	**Firmic u.**	**Fusob ac.**	**Planct o.**	**Prot eob.**	**Spiroc h.**	**Synerg i.**	**Teneri c.**	**Verruc .**	**Other**	
Blattella germanica		515F-907R	Illumina MiSeq	No data	foregut	0	13	48	0.7	0.9	34	0	0	0.5	0	3.2	(Zhang and Yang 2019)
					midgut	0	7.2	34	2.8	0.5	54	0	0	0.2	0	2.0	
					hindgut	0	50	29	3.2	4.9	9.3	0	0	0.4	0	2.8	
Byrsotria fumigata		343F-784R	Illumina MiSeq	DictDb v.3.0	crop	13	1.6	72	1.1	0.1	11	0.5	0	0.1	0	0.6	(Lampert et al. 2019)
					midgut	3.2	29	44	0.5	0.2	21	0.7	0	0.1	0	1.3	
					hindgut	3.8	27	55	1.0	2.1	7.7	0.5	0.3	0.1	0	2.5	
Ergaula capucina		343F-784R	Illumina MiSeq	DictDb v.3.0	crop	21	14	52	0	0.2	10	0.2	0	0.4	0.1	2.1	(Lampert et al. 2019)
					midgut	6.9	26	49	0.1	0.6	14	0.5	0.1	1.2	0	1.6	
					hindgut	5.5	34	51	0	0.7	4	0.1	0.9	0.3	0	3.5	
Panchlora sp.	A	V6-V8 region	454 Roche pyroseq	NCBI 16S rRNA	foregut	1.2	1.6	7.7	0.03	0.007	89	0	0.06	0	0.007	0.4	(Gontang et al. 2017)
					midgut	2.8	1.7	33	0.2	0.007	62	0	0.06	0	0.01	0.23	
					hindgut	1.2	18	20	1.2	0	51	0.09	2.7	0	0.1	5.7	
	B	V6-V8 region	454 Roche pyroseq	NCBI 16S rRNA	foregut	0.1	0.3	4.6	0.1	0	93	0.3	0	0.60	0	1.0	(Gontang et al. 2017)
					midgut	0.1	0.9	39	0.7	0	54	1.0	0.002	1.9	0	2.4	
					hindgut	1.8	29	19	1.4	0.1	44	0.8	0.8	1.7	0.1	1.3	
Panesthia angustipennis		V3-V4 region	454 Roche pyroseq	DictDb	crop	4.0	5.4	5.9	0.01	0.5	65	1.0	0.1	0	3.5	14.6	(Bauer et al. 2015)
					midgut	0.33	15	57	4.8	0.6	18	0.05	0.5	0	0.9	2.8	
					hindgut	1.0	23	42	0.2	3.4	3.3	0.7	13	0	3.9	3.2	
Pycnoscelus surinamensis		343F-784R	Illumina MiSeq	DictDb v.3.0	crop	16	18	23	0.1	0.7	40	0.4	0.2	0.2	0.1	1.3	(Lampert et al. 2019)
					midgut	0.9	22	24	2.9	0.6	10	37	0.7	0.1	0	1.8	
					hindgut	1.5	45	17	0.2	1.6	32	0.4	0.4	0.1	0.2	1.6	
Salganea esakii		V3-V4 region	454 Roche pyroseq	DictDb	crop	1.9	4.7	27	0.04	1.9	38	0	0.2	0	9.4	17	(Bauer et al. 2015)
					midgut	1.4	3.1	64	6.1	0.5	16	0.01	0.04	0	1.2	7.7	
					hindgut	0.7	31	41	0.2	2.2	4.2	1.1	5.2	0	1.5	12.9	

The data presented here was either extracted directly from the supplementary information of the published papers, or was obtained from the authors, and in some cases the data was calculated from the tables and plots in the original publications.

References

Abdolmaleki Z, Mashak Z, Safarpoor Dehkordi F (2019) Phenotypic and genotypic characterization of antibiotic resistance in the methicillin-resistant *Staphylococcus aureus* strains isolated from hospital cockroaches. Antimicrob Resist Infect Control 8(1):54. doi:10.1186/s13756-019-0505-7

Agbodaze S (1989) Cockroaches (*Periplaneta americana*) as carriers of agents of bacterial diarrhoea in Accra, Ghana. Centr Afr J Med 35(9):484-486.

Akbari S, Oshaghi MA, Hashemi-Aghdam SS, Hajikhani S, Oshaghi G, Shirazi MH (2015) Aerobic bacterial community of American cockroach *Periplaneta americana*, a step toward finding suitable paratransgenesis candidates. J Arthropod-Borne Dis 9(1):35.

Akinjogunla OJ, Odeyemi A, Udoinyang E (2012) Cockroaches (*Periplaneta americana* and *Blattella germanica*): reservoirs of multi drug resistant (MDR) bacteria in Uyo, Akwa Ibom State. Sci J Biol Sci 1(2):19.

Al-Fattly H, Al-Aridhi HS (2014) Antibiotic resistant bacteria associated with the cockroach *Periplaneta americana* in Al-Diwaniya city/Iraq. Int J Adv Res 2:709-714.

Alcamo IE, Frishman AM (1980) The microbial flora of field-collected cockroaches and other arthropods. J Environ Health 42(5):263-266.

Bagde U, Gopi U, Prasad R (2013) Isolation and characterization of gut-associated microbes in cockroach. Afr J Microbiol Res 7(9):2034-2039.

Bauer E, Lampert N, Mikaelyan A, Köhler T, Maekawa K, Brune A (2015) Physicochemical conditions, metabolites and community structure of the bacterial microbiota in the gut of wood-feeding cockroaches (Blaberidae: Panesthiinae). FEMS Microbiol Ecol 91(2):1-14. doi: 10.1093/femsec/fiu028

Berlanga M, Paster BJ, Guerrero R (2009) The taxophysiological paradox: changes in the intestinal microbiota of the xylophagous cockroach *Cryptocercus punctulatus* depending on the physiological state of the host. Int Microbiol 12(4):227-236.

Bertino-Grimaldi D, Medeiros MN, Vieira RP, Cardoso AM, Turque AS, Silveira CB, Albano RM, Bressan-Nascimento S, Garcia ES, de Souza W (2013) Bacterial community composition shifts in the gut of *Periplaneta americana* fed on different lignocellulosic materials. SpringerPlus 2(1):609. doi:10.1186/2193-1801-2-609

Bouamamaa L, Sorlozano A, Laglaoui A, Lebbadi M, Aarab A, Gutierrez J (2010) Antibiotic resistance patterns of bacterial strains isolated from *Periplaneta americana* and *Musca domestica* in Tangier, Morocco. J Infect Dev Countr 4(4):194-201. doi:10.3855/jidc.336

Burgess N, McDermott S, Whiting J (1973) Aerobic bacteria occurring in the hind-gut of the cockroach, *Blatta orientalis*. Epidemiol Infect 71(1):1-7. doi:10.1017/s0022172400046155

Chen Z, Ou P, Liu L, Jin X (2020) Anti-MRSA Activity of Actinomycin X2 and Collismycin A Produced by *Streptomyces globisporus* WA5-2-37 From the Intestinal Tract of American Cockroach (*Periplaneta americana*). Front Microbiol 11:555. doi:10.3389/fmicb.2020.00555

Cloarec A, Rivault C, Fontaine F, Le Guyader A (1992) Cockroaches as carriers of bacteria in multi-family dwellings. Epidemiol Infect 109(3):483-490

Cruden DL, Markovetz A (1987) Microbial ecology of the cockroach gut. Ann Rev Microbiol 41(1):617-643. doi:10.1146/annurev.mi.41.100187.003153

Cruden DL, Markovetz A (1979) Carboxymethyl cellulose decomposition by intestinal bacteria of cockroaches. Appl Environ Microbiol 38(3):369-372.

Dietrich C, Köhler T, Brune A (2014) The Cockroach Origin of the Termite Gut Microbiota: Patterns in Bacterial Community Structure Reflect Major Evolutionary Events. Appl Environ Microbiol 80(7):2261-2269. doi:10.1128/aem.04206-13

Dugas J, Zurek L, Paster B, Keddie B, Leadbetter E (2001) Isolation and characterization of a *Chryseobacterium* strain from the gut of the American cockroach, *Periplaneta americana*. Arch Microbiol 175(4):259-262. doi:10.1007/s002030000243

Elgderi R, Ghenghesh K, Berbash N (2006) Carriage by the German cockroach (Blattella germanica) of multiple-antibiotic-resistant bacteria that are potentially pathogenic to humans, in hospitals and households in Tripoli, Libya. Ann Trop Med Parasitol 100(1):55-62.

Elyasigomari A, Keshavarzi D, Ahmed Yusuf M, Hassanzadeh A, Marvi N, Shahriari-Namadi M, Nassiri Z (2017) Isolation of bacteria from the digestive tract of *Periplaneta americana* and *Polyphaga aegyptiaca* (Blattodea: Blattidae) in Khuzestan Province, Southwestern Iran. Orient Insects 51(4):345-352. doi:10.1080/00305316.2017.1311811

Fakoorziba MR, Shahriari-Namadi M, Moemenbellah-Fard MD, Hatam GR, Azizi K, Amin M, Motevasel M (2014) Antibiotics susceptibility patterns of bacteria isolated from American and German cockroaches as potential vectors of microbial pathogens in hospitals. Asian Pac J Trop Dis 4:S790-S794. doi:10.1016/S2222-1808(14)60728-3

Fathpour H, Emtiazi G, Ghasemi E (2003) Cockroaches as reservoirs and vectors of drug resistant *Salmonella* spp. Iranian Biomed J 7(1):35-38.

Feinberg L, Jorgensen J, Haselton A, Pitt A, Rudner R, Margulis L (1999) Arthromitus (*Bacillus cereus*) symbionts in the cockroach *Blaberus giganteus*: dietary influences on bacterial development and population density. Symbiosis 27(2):109-123.

Feizhaddad M-H, Kassiri H, Sepand M-R, Ghasemi F (2012) Bacteriological survey of American cockroaches in hospitals. Middle East J Sci Res 12(7):985-989.

Foglesong M, Cruden D, Markovetz A (1984) Pleomorphism of fusobacteria isolated from the cockroach hindgut. J Bacteriol 158(2):474-480.

Fotedar R, Shriniwas UB, Verma A (1991) Cockroaches (*Blattella germanica*) as carriers of microorganisms of medical importance in hospitals. Epidemiol Infect 107(1):181-187. doi:10.1017/s0950268800048809

García F, Notario M, Cabanás J, Jordano R, Medina L (2012) Incidence of bacteria of public health interest carried by cockroaches in different food-related environments. J Med Entomol 49(3):1481-1484.

Gliniewicz A, Czajka E, Laudy A, Kochman M, Grzegorzak K, Ziółkowska K, Sawicka B, Stypulkowska-Misiurewicz H, Pancer K (2003) German cockroaches (*Blattella germanica* L.) as a potential source of pathogens causing nosocomial infections. Indoor Built Environ 12(1-2):55-60.

Gontang EA, Aylward FO, Carlos C, del Rio TG, Chovatia M, Fern A, Lo C-C, Malfatti SA, Tringe SG, Currie CR (2017) Major changes in microbial diversity and community composition across gut sections of a juvenile *Panchlora* cockroach. PLoS One 12(5). doi:10.1371/journal.pone.0177189

Gonzalez-Astudillo V, Bustamante-Rengifo JA, Bonilla Á, Lehmicke AJJ, Castillo A, Astudillo-Hernández M (2015) Synanthropic Cockroaches (Blattidae: *Periplaneta* spp.) Harbor Pathogenic *Leptospira* in Colombia. J Med Entomol 53(1):177-182. doi:10.1093/jme/tjv172

Hoffman GL (1953) *Streptomyces leidnematis* n. sp., growing on Two Species of Nematodes of the Cockroach. Trans Am Microscop Soc 72(4):376-378. doi:10.2307/3223487

Huang YH, Wang XJ, Zhang F, Huo XB, Fu RS, Liu JJ, Sun WB, Kang DM, Jing X (2013) The identification of a bacterial strain BGI-1 isolated from the intestinal flora of *Blattella germanica*, and its anti-entomopathogenic fungi activity. J Econ Entomol 106(1):43-9. doi:10.1603/ec12120

Iboh C, Etim L, Abraham J, Ajang R (2014) Bacteria and parasites infestation of cockroaches in a developing community, South Eastern, Nigeria. Int J Bacteriol Res 2(5):45-48.

Isaac C, Orue PO, Iyamu MI, Ehiaghe JI, Isaac O (2014) Comparative analysis of pathogenic organisms in cockroaches from different community settings in Edo State, Nigeria. Kor J Parasitol 52(2):177.

Islam A, Nath AD, Islam K, Islam S, Chakma S, Hossain MB, Al-Faruq A, Hassan MM (2016) Isolation, identification and antimicrobial resistance profile of *Staphylococcus aureus* in Cockroaches (*Periplaneta americana*). J Adv Vet Anim Res 3(3):221-228.

Jalil N, Amir K, Hasan M-KS, Mahdi M, Monireh M, Atefeh B (2012) Cockroaches' bacterial infections in wards of hospitals, Hamedan city, west of Iran. Asian Pac J Trop Dis 2(5):381-4

Jeffery J, Sulaiman S, Oothuman P, Vellayan S, Zainol-Ariffin P, Paramaswaran S, Razak A, Muslimin M, Kamil-Ali O, Rohela M (2012) Domiciliary cockroaches found in restaurants in five zones of Kuala Lumpur Federal Territory, peninsular Malaysia. Trop Biomed 29(1):180-186

J Jennings EC, Korthauer MW, Hamilton TL, Benoit JB (2019) Matrotrophic viviparity constrains microbiome acquisition during gestation in a live-bearing cockroach, *Diploptera punctata*. Ecol Evol 9(18):10601-10614. doi:10.1002/ece3.5580

Kakumanu ML, Maritz JM, Carlton JM, Schal C (2018) Overlapping Community Compositions of Gut and Fecal Microbiomes in Lab-Reared and Field-Collected German Cockroaches. Appl Environ Microbiol 84(17):e01037-18. doi:10.1128/aem.01037-18

Kane MD, Breznak JA (1991) Effect of host diet on production of organic acids and methane by cockroach gut bacteria. Appl Environ Microbiol 57(9):2628-2634.

Karimi Zarchi AA, Vatani H (2009) A Survey on Species and Prevalence Rate of Bacterial Agents Isolated from Cockroaches in Three Hospitals. Vector Borne Zoonotic Dis 9(2):197-200. doi:10.1089/vbz.2007.0230

Kassiri H, Kasiri A, Quaderi A (2014) Detection and prevalence rate of American cockroaches (*Periplaneta americana*) bacterial infections in human dwellings. Arch Clin Infect Dis 9(4).

Lampert N, Mikaelyan A, Brune A (2019) Diet is not the primary driver of bacterial community structure in the gut of litter-feeding cockroaches. BMC Microbiol 19(1):238. doi:10.1186/s12866-019-1601-9

Le Guyader A, Rivault C, Chaperon J (1989) Microbial organisms carried by brown-banded cockroaches in relation to their spatial distribution in a hospital. Epidemiol Infect 102(3):485-492. doi: 10.1017/s095026880003020x

Leibovitz A (1951) The cockroach, *Periplaneta americana*, as a vector of pathogenic organisms: I. The acid-fast organisms: Preliminary Report. Boletín de la Oficina Sanitaria Panamericana (OSP); 30 (1), ene 1951.

Leite-Vicente CS, Ozawa S, Hasegawa K (2018) The composition of hindgut microbiota of *Periplaneta japonica* in the presence of thelastomatid parasitic nematodes. Nematol Res 48(1):19-26.

Loucif L, Cherak Z, Chamlal N, Bendjama E, Gacemi-Kirane D, Grainat N, Rolain J-M (2017) First Detection of VIM-2 Metallo-β-Lactamase-Producing *Pseudomonas putida* in *Blattella germanica* Cockroaches in an Algerian Hospital. Antimicrob Ag Chemother 61(8):e00357-17. doi:10.1128/aac.00357-17

Margulis L, Jorgensen JZ, Dolan S, Kolchinsky R, Rainey FA, Lo S-C (1998) The Arthromitus stage of *Bacillus cereus*: Intestinal symbionts of animals. Proc Natl Acad Sci USA 95(3):1236-1241. doi:10.1073/pnas.95.3.1236

Menasria T, Moussa F, El-Hamza S, Tine S, Megri R, Chenchouni H (2014) Bacterial load of German cockroach (*Blattella germanica*) found in hospital environment. Pathog Glob Health 108(3):141-147. doi:10.1179/2047773214Y.0000000136

Menasria T, Tine S, Mahcene D, Benammar L, Megri R, Boukoucha M, Debabza M (2015) External Bacterial Flora and Antimicrobial Susceptibility Patterns of *Staphylococcus* spp. and *Pseudomonas* spp. Isolated from Two Household Cockroaches, *Blattella germanica* and *Blatta orientalis*. Biomed Environ Sci 28(4):316-320. doi:10.3967/bes2015.045

Mikaelyan A, Köhler T, Lampert N, Rohland J, Boga H, Meuser K, Brune A (2015) Classifying the bacterial gut microbiota of termites and cockroaches: a curated phylogenetic reference database (DictDb). Syst Appl Microbiol 38(7):472-482. doi: 10.1016/j.syapm.2015.07.004

Mikaelyan A, Thompson CL, Hofer MJ, Brune A (2016) Deterministic Assembly of Complex Bacterial Communities in Guts of Germ-Free Cockroaches. Appl Environ Microbiol 82(4):1256-1263 doi:10.1128/aem.03700-15.

Mpuchane S, Allotey J, Matsheka I, Simpanya M, Coetzee S, Jordaan A, Mrema N, Gashe B (2006a) Carriage of micro-organisms by domestic cockroaches and implications on food safety. Int J Trop Insect Sci26(3):166-175.

Mpuchane S, Matsheka IM, Gashe BA, Allotey J, Murindamombe G, Mrema N (2006b) Microbiological studies of cockroaches from three localities in Gaborone, Botswana. Afr J Food Agric Nutr Develop 6(2).

Naher A, Afroz S, Hamid S (2018) Cockroach associated foodborne pathogens: Distribution and antibiogram. Bangladesh Med Res Counc Bull 44:30. doi:10.3329/bmrcb.v44i1.36802

Oliva GR, Díaz C, González OF, Martínez M, Fernández C, Cordoví R, Lago PM, Herrera N (2010) *Blatella germanica* as a possible cockroach vector of micro-organisms in a hospital. J Hosp Infect74(1):93-95. doi:10.1016/j.jhin.2009.09.002

Oothuman P, Jeffery J, Aziz AHA, Bakar EA, Jegathesan M (1989) Bacterial pathogens isolated from cockroaches trapped from paediatric wards in peninsular Malaysia. Trans R Soc Trop Med Hyg 83(1):133-135. doi:10.1016/0035-9203(89)90739-6

Pai HH, Chen WC, Peng CF (2005) Isolation of bacteria with antibiotic resistance from household cockroaches (*Periplaneta americana* and *Blattella germanica*). Acta Trop 93(3):259-265. doi:10.1016/j.actatropica.2004.11.006

Pai HH, Chen WC, Peng CF (2003) Isolation of non-tuberculous mycobacteria from hospital cockroaches (*Periplaneta americana*). J Hosp Infect 53(3):224-8. doi:10.1053/jhin.2002.1355

Pérez-Cobas AE, Maiques E, Angelova A, Carrasco P, Moya A, Latorre A (2015) Diet shapes the gut microbiota of the omnivorous cockroach *Blattella germanica*. FEMS Microbiol Ecol 91(4):fiv022. doi: 10.1093/femsec/fiv022

Pietri JE, Tiffany C, Liang D (2018) Disruption of the microbiota affects physiological and evolutionary aspects of insecticide resistance in the German cockroach, an important urban pest. PLoS One 13(12):e0207985. doi: 10.1371/journal.pone.0207985

Prado MA, Gir E, Pereira MS, Reis C, Pimenta FC (2006) Profile of antimicrobial resistance of bacteria isolated from cockroaches (*Periplaneta americana*) in a Brazilian health care institution. Braz J Infect Dis 10(1):26-32. doi: 10.1590/S1413-86702006000100006

Rampal L, Oothuman P, Jeffery J, Daud M, Shekhar C, Senan P, Eow L, Suboh Y, Ahmad Z (1983) Bacterial pathogens from the intestinal tracts of various species of cockroaches. Med J Malaysia 38(2):104-107.

Ratcliffe NA, Rowley AF (1984) Opsonic activity of insect hemolymph. In: Cheng TC (ed) Invertebrate Blood: Cells and Serum Factors. Springer US, Boston, MA, pp 187-204.

Richards C, Otani S, Mikaelyan A, Poulsen M (2017) *Pycnoscelus surinamensis* cockroach gut microbiota respond consistently to a fungal diet without mirroring those of fungus-farming termites. PLoS One 12(10):e0185745. doi:10.1371/journal.pone.0185745

Robertson AR (2007) The Isolation and Characterization of the Microbial Flora in the Alimentary Canal of *Gromphadorhina portentosa* Based on rDNA Sequences. East Tennesee State University. Electronic Theses and Dissertations. Paper 2069. https://dc.etsu.edu/etd/2069

Rosas T, García-Ferris C, Domínguez-Santos R, Llop P, Latorre A, Moya A (2018) Rifampicin treatment of *Blattella germanica* evidences a fecal transmission route of their gut microbiota. FEMS Microbiol Ecol 94(2):fiy002. doi:10.1093/femsec/fiy002

Roth L, Willis E (1960) The biotic associations of cockroaches. The Smithsonian Institute, Washington, D.C.

Rueger ME, Olson TA (1969) An Epizootic of *Serratia marcescens* (Enterobacteriaceae) in *Periplaneta fuliginosa* (Blattaria) and A Suggested Method for Control. J Med Entomol 6(2):190-190.

Saitou K, Furuhata K, Kawakami Y, Fukuyama M (2009) Isolation of *Pseudomonas aeruginosa* from cockroaches Captured in hospitals in Japan, and their antibiotic susceptibility. Biocontrol Sci 14(4):155-9. doi:10.4265/bio.14.155

Salehzadeh A, Tavacol P, Mahjub H (2007) Bacterial, fungal and parasitic contamination of cockroaches in public hospitals of Hamadan, Iran. J Vect Borne Dis 44(2):105-110.

Sayyad S, Vahabi A, Vahabi B, Sayyadi M, Sahne SH (2016) Investigation of bacteriological infections of the American Cockroaches in Paveh City, Kermanshah Province. Materia socio-medica 28(1):17 .

Schauer C, Thompson C, Brune A (2014) Pyrotag sequencing of the gut microbiota of the cockroach *Shelfordella lateralis* reveals a highly dynamic core but only limited effects of diet on community structure. PLoS One 9(1):e85861. doi:10.1371/journal.pone.0085861

Sharma S, Prasad RK, Chatterjee S, Sharma A, Vairale MG, Yadav KK (2019) Characterization of *Bacillus* Species with Keratinase and Cellulase Properties Isolated from Feather Dumping Soil and Cockroach Gut. Proc Natl Acad Sci, India Sect B: Biol Sc 89(3):1079-1086.

Sravanthi T, Tushar L, Sasikala C, Ramana CV (2016) *Alkalispirochaeta cellulosivorans* gen. nov., sp. nov., a cellulose-hydrolysing, alkaliphilic, halotolerant bacterium isolated from the gut of a wood-eating cockroach (*Cryptocercus punctulatus*), and reclassification of four species of *Spirochaeta* as new combinations within *Alkalispirochaeta* gen. nov. Int J Syst Evol Microbiol 66(4):1612-1619. doi:10.1099/ijsem.0.000865

Strand MA, Brooks MA (1977) Pathogens of Blattidae (cockroaches). Bull World Health Organ 55(1):289-296.

Tachbele E, Erku W, Gebre-Michael T, Ashenafi M (2006) Cockroach-associated food-borne bacterial pathogens from some hospitals and restaurants in Addis Ababa, Ethiopia: Distribution and antibiograms. J Rural Trop Pub Health 5(1):34-41.

Tegtmeier D, Belitz A, Radek R, Heimerl T, Brune A (2018) *Ereboglobus luteus* gen. nov. sp. nov. from cockroach guts, and new insights into the oxygen relationship of the genera Opitutus and Didymococcus (Verrucomicrobia: Opitutaceae). Syst Appl Microbiol 41(2):101-112. doi:10.1016/j.syapm.2017.10.005

Tegtmeier D, Riese C, Geissinger O, Radek R, Brune A (2016a) *Breznakia blatticola* gen. nov. sp. nov. and *Breznakia pachnodae* sp. nov., two fermenting bacteria isolated from insect guts, and emended description of the family Erysipelotrichaceae. Syst Appl Microbiol 39(5):319-329. doi:https://doi.org/10.1016/j.syapm.2016.05.003

Tegtmeier D, Thompson CL, Schauer C, Brune A (2016b) Oxygen affects gut bacterial colonization and metabolic activities in a gnotobiotic cockroach model. Appl Environ Microbiol 82(4):1080-1089. doi:10.1128/aem.03130-15

Tetteh-Quarcoo PB, Donkor ES, Attah SK, Duedu KO, Afutu E, Boamah I, Olu-Taiwo M, Anim-Baidoo I, Ayeh-Kumi PF (2013) Microbial carriage of cockroaches at a tertiary care hospital in Ghana. Environ Health Insights 7:EHI-S12820.

Tilahun B, Worku B, Tachbele E, Terefe S, Kloos H, Legesse W (2012) High load of multi-drug resistant nosocomial neonatal pathogens carried by cockroaches in a neonatal intensive care unit at Tikur Anbessa specialized hospital, Addis Ababa, Ethiopia. Antimicrob Resist Infect Control 1(1):12. doi:10.1186/2047-2994-1-12

Tinker KA, Ottesen EA (2016) The core gut microbiome of the American cockroach, *Periplaneta americana*, is stable and resilient to dietary shifts. Appl Environ Microbiol 82(22):6603-6610. doi:10.1128/aem.01837-16

Tinker KA, Ottesen EA (2020) Phylosymbiosis across Deeply Diverging Lineages of Omnivorous Cockroaches (Order Blattodea). Appl Environ Microbiol 86(7):e02513-19 doi:10.1128/aem.02513-19.

Vahabi A, Shemshad K, Mohammadi P, Sayyadi M, Shemshad M, Rafinejad J (2011) Microbiological study of domestic cockroaches in human dwelling localities. African J Microbiol Res 5(31):5790-5792.

Vazirianzadeh B, Dehghani R, Mehdinejad M, Sharififard M, Nasirabadi N (2014) The first report of drug resistant bacteria isolated from the brown-banded cockroach, Supella longipalpa, in ahvaz, south-western Iran. J Arthropod-Borne Dis 8(1):53.

Vazirianzadeh B, Manijeh M, Dehghani R (2009) Identification of bacteria which possible transmitted by *Polyphaga aegyptica* (Blattodea: Blattidae) in the region of Ahvaz, SW Iran. Jundishapur J Microbiol 2(1):36-40.

Vera-Ponce de León A, Jahnes BC, Duan J, Camuy-Vélez LA, Sabree ZL (2020) Cultivable, Host-Specific Bacteroidetes Symbionts Exhibit Diverse Polysaccharolytic Strategies. Appl Environ Microbiol 86(8):e00091-20. doi:10.1128/aem.00091-20

Wannigama DL, Dwivedi R, Zahraei-Ramazani A (2014) Prevalence and antibiotic resistance of Gram-negative pathogenic bacteria species isolated from *Periplaneta americana* and *Blattella germanica* in Varanasi, India. J Arthropod-borne Dis 8(1):10-20.

Zhang F, Huang YH, Liu SZ, Zhang L, Li BT, Zhao XX, Fu Y, Liu JJ, Zhang XX (2013) *Pseudomonas reactans*, a bacterial strain isolated from the intestinal flora of *Blattella germanica* with anti-*Beauveria bassiana* activity. Environ Entomol 42(3):453-9. doi:10.1603/en12347

Zhang F, Sun XX, Zhang XC, Zhang S, Lu J, Xia YM, Huang YH, Wang XJ (2018) The interactions between gut microbiota and entomopathogenic fungi: a potential approach for biological control of *Blattella germanica* (L.). Pest Manag Sci 74(2):438-447. doi:10.1002/ps.4726

Zhang F, Yang R (2019) Life history and functional capacity of the microbiome are altered in beta-cypermethrin-resistant cockroaches. Int J Parasitol 49(9):715-723. doi:10.1016/j.ijpara.2019.04.006
